# Biochemical and Structural Characteristics, Gene Regulation, Physiological, Pathological and Clinical Features of Lipocalin-Type Prostaglandin D_2_ Synthase as a Multifunctional Lipocalin

**DOI:** 10.3389/fphys.2021.718002

**Published:** 2021-10-22

**Authors:** Yoshihiro Urade

**Affiliations:** ^1^Center for Supporting Pharmaceutical Education, Daiichi University of Pharmacy, Fukuoka, Japan; ^2^Isotope Science Center, The University of Tokyo, Tokyo, Japan

**Keywords:** sleep, food intake, adipogenesis, innate immunity, inflammation, beta-trace protein, amyloid-beta chaperon, reproduction

## Abstract

Lipocalin-type prostaglandin (PG) D_2_ synthase (L-PGDS) catalyzes the isomerization of PGH_2_, a common precursor of the two series of PGs, to produce PGD_2_. PGD_2_ stimulates three distinct types of G protein-coupled receptors: (1) D type of prostanoid (DP) receptors involved in the regulation of sleep, pain, food intake, and others; (2) chemoattractant receptor-homologous molecule expressed on T helper type 2 cells (CRTH2) receptors, in myelination of peripheral nervous system, adipocyte differentiation, inhibition of hair follicle neogenesis, and others; and (3) F type of prostanoid (FP) receptors, in dexamethasone-induced cardioprotection. L-PGDS is the same protein as β-trace, a major protein in human cerebrospinal fluid (CSF). L-PGDS exists in the central nervous system and male genital organs of various mammals, and human heart; and is secreted into the CSF, seminal plasma, and plasma, respectively. L-PGDS binds retinoic acids and retinal with high affinities (Kd < 100 nM) and diverse small lipophilic substances, such as thyroids, gangliosides, bilirubin and biliverdin, heme, NAD(P)H, and PGD_2_, acting as an extracellular carrier of these substances. L-PGDS also binds amyloid β peptides, prevents their fibril formation, and disaggregates amyloid β fibrils, acting as a major amyloid β chaperone in human CSF. Here, I summarize the recent progress of the research on PGD_2_ and L-PGDS, in terms of its “molecular properties,” “cell culture studies,” “animal experiments,” and “clinical studies,” all of which should help to understand the pathophysiological role of L-PGDS and inspire the future research of this multifunctional lipocalin.

## Introduction

In 1985, I purified lipocalin-type prostaglandin (PG) D_2_ synthase (L-PGDS) from rat brain as a prostaglandin H_2_ (PGH_2_) D-isomerase (EC:5.3.99.2), that catalyzes the isomerization of a 9,11-endoperoxide group of PGH_2_, a common intermediate of the two series of prostanoids, to produce prostaglandin D_2_ (PGD_2_) with 9-hydroxy and 11-keto groups (Urade et al., 1985). The cDNA cloning of L-PGDS demonstrated that the amino acid sequence of L-PGDS has the homology with members of the lipocalin family which is composed of a variety of secretory proteins that bind and transport lipophilic small substances, such as β-lactoglobulin, α2-urinary globulin, placental protein 14, and α1-microglobulin (Urade and Hayaishi, 2000a). L-PGDS possesses a typical lipocalin fold of β-barrel with a central hydrophobic cavity, the retinoid-binding activity similar to other lipocalins, and the chromosomal gene structure with the same numbers and sizes of exons and phase of splicing of introns as those of other lipocalins (Urade and Hayaishi, 2000a).

Those studies open a new field of the lipocalin research, because that L-PGDS is the first lipocalin characterized as an enzyme among members of the lipocalin family and that L-PGDS produces an important bioactive lipid mediator PGD_2_. PGD_2_ plays important roles in the regulation of a variety of patho-physiological functions, such as sleep, pain, food intake in the central nervous system (CNS), inflammation and innate immunity, diabetes, cardiovascular functions and also in the reproduction systems.

I published several reviews of L-PGDS and PGD_2_ (Urade and Hayaishi, 2000a, b, 2011; Urade and Eguchi, 2002; Smith et al., 2011). In this article, I classify the reports concerning with L-PGDS mainly after publication of those reviews and summarize the new finding of each section as follows:

1) Biological function of prostaglandin D_2_ produced by lipocalin-type prostaglandin D_2_ synthase,
2) Ligand binding properties of lipocalin-type prostaglandin D_2_ synthase as an extracellular transporter,
3) Structural characterization of lipocalin-type prostaglandin D_2_ synthase by nuclear magnetic resonance (NMR) and X-ray crystallography,
4) Cell culture studies of lipocalin-type prostaglandin D_2_ synthase,
5) Mammalian experiments for the study of lipocalin-type prostaglandin D_2_ synthase,
6) Pharmacokinetic analyses and functionalization with recombinant lipocalin-type prostaglandin D_2_ synthase,
7) Studies of nonmammalian orthologs of lipocalin-type prostaglandin D_2_ synthase,
8) Clinical studies of pathophysiological function of lipocalin-type prostaglandin D_2_ synthase,
9) Future subjects.

## Biological Function of Prostaglandin D_2_ Produced by Lipocalin-Type Prostaglandin D_2_ Synthase

Supplementary Table 1 summarizes the research history of L-PGDS and PGD_2_. PGD_2_ was originally discovered as a by-product of biosynthesis of prostaglandin E_2_ (PGE_2_) and prostaglandin F_2__α_ (PGF_2α_). Both PGE_2_ and PGF_2α_ exhibit potent activities on the smooth muscle contraction, whereas PGD_2_ does not show the strong smooth muscle contractile activity. Therefore, the physiological function of PGD_2_ was not extensively investigated until the discovery of potent action of PGD_2_ on the regulation of inflammation and sleep.

In the early 1980s, PGD_2_ was found to be a major prostaglandin produced in the brain of various mammals (Narumiya et al., 1982) including humans (Ogorochi et al., 1984) and to induce sleep after administration into the brain of freely moving rats (Ueno et al., 1982) and monkeys (Onoe et al., 1988). From those findings, the biochemical and neurological studies of PGD_2_ in the CNS were accelerated. In 1979, other type of PGD_2_ synthase, hematopoietic PGD_2_ synthase (H-PGDS), was purified from rat spleen (Christ-Hazelhof and Nugteren, 1979). Comparisons of L-PGDS with H-PGDS revealed that these two enzymes are quite different proteins from each other (Urade et al., 1987), i.e., L-PGDS is a member of the lipocalin family (Nagata et al., 1991) and H-PGDS is the first identified invertebrate ortholog of sigma class of glutathione *S*-transferase (Kanaoka et al., 1997; Kanaoka and Urade, 2003). L-PGDS and H-PGDS have been evolved from different origins to acquire the same catalytic ability, being new examples of functional convergence (Urade and Eguchi, 2002; Smith et al., 2011).

Supplementary Table 2 summarizes the catalytic, molecular and genetic properties of human L-PGDS and H-PGDS. We isolated cDNAs and chromosomal genes of L-PGDS (*L-Pgds* or *Ptgds*; Urade et al., 1989; Igarashi et al., 1992) and H-PGDS (*Hpgds*; Kanaoka et al., 1997, 2000), and then determined X-ray crystallographic structures of the recombinant proteins of L-PGDS and H-PGDS expressed in *E. coli* (Kanaoka et al., 1997; Kumasaka et al., 2009), respectively. The inhibitors selective to L-PGDS, SeCl_4_ and AT56 (Irikura et al., 2009), and to H-PGDS, HQL79 (Aritake et al., 2006), TFC007 (Nabea et al., 2011) and TAS204 (Urade, 2016) were found. We also generated KO mice of *L-Pgds* and *Hpgds* genes (Eguchi et al., 1999; Park et al., 2007), respectively, human enzyme-overexpressing transgenic (TG) mice (Fujitani et al., 2002, 2010), respectively, and flox mice used for conditional KO mice (Kaneko et al., 2012; Nakamura et al., 2017), respectively. In double KO mice of *L-Pgds* and *Hpgds* genes, the production of PGD_2_ in the brain and other tissues is almost undetectable, indicating that these two enzymes are major components responsible for the biosynthesis of PGD_2_ in our body (Kaushik et al., 2014).

**FIGURE 1:**
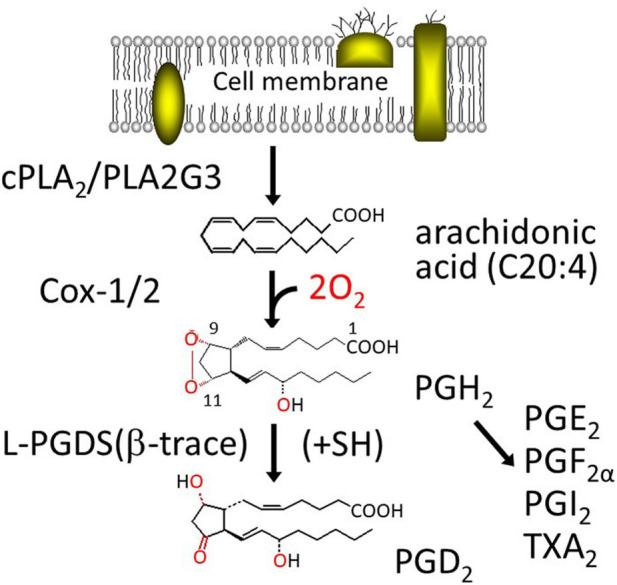
Biosynthesis pathway of PGD_2_.

Figure 1 shows the biosynthesis of PGD_2_. PGD_2_ is produced from arachidonic acid (C20:4), a major polyunsaturated fatty acid in our body, integrated in C2 position of phospholipids. Once cells are stimulated by various hormones, cytokines, and other signals, arachidonic acid is released from phospholipids by the action of cytosolic phospholipase A_2_ (cPLA_2_) or group III phospholipase A_2_ (PLA2G3). A part of the released arachidonic acid is oxygenated by cyclooxygenase (Cox)-1 or 2 (PGH_2_ synthase-1 or -2, these genes are *ptgs* or *ptgs2*, respectively) to produce PGH_2_, a common intermediate of the two series of PGs, PGD_2_, PGE_2_, PGF_2α_, PGI_2_ (prostacyclin) and thromboxane (TX) A_2_, in which 2 indicates the number of unsaturated C=C bond. PGH_2_ is converted by L-PGDS or H-PGDS to PGD_2_, in the presence of exogenous sulfhydryl compounds, most likely a reduced form of glutathione within the cells. Both Cox-1 and -2 are microsomal membrane-binding enzymes and produce PGH_2_ within the cells. PGH_2_ is chemically unstable in aqueous solution with a t_1/2_ of several min to degrade a mixture of PGE_2_ and PGD_2_ at a ratio of 2:1. Therefore, it is unlikely that L-PGDS in the extracellular space interacts with PGH_2_ to produce PGD_2_ selectively. As L-PGDS binds a variety of hydrophobic substances, such as retinoids and thyroids, L-PGDS in the extracellular space may act as an extracellular transporter for these hydrophobic ligands.

Prostaglandin D_2_ stimulates three distinct types of G-protein coupled receptors (Supplementary Table 3). One is D type of prostanoid (DP) receptors (also abbreviated as DP_1_) coupled with Gs-protein to increase intracellular cAMP levels (Hirata et al., 1994). DP receptors are involved in the regulation of sleep (Qu et al., 2006), pain (Eguchi et al., 1999), food intake (Ohinata et al., 2008) and others. Two is chemoattractant receptor-homologous molecule expressed on T helper type 2 cells (CRTH2) receptors (also abbreviated as DP_2_, previously known as GPR44 of an orphan receptor, or CD294) coupled with Gi-protein to decrease intracellular cAMP levels (Nagata et al., 1999; Hirai et al., 2001). CRTH2 receptors are involved in myelination of peripheral nervous system (Trimarco et al., 2014), adipocyte differentiation (Wakai et al., 2017), inhibition of hair follicle neogenesis (Nelson et al., 2013) and others. Three is F type of prostanoid (FP) receptors (Abramovitz et al., 1994) coupled with Gq-protein to increase intracellular Ca^2+^ concentrations. FP receptors are activated by either PGD_2_ or PGF_2α_ at almost the same binding affinities and involved in protection of the heart against ischemia-reperfusion injury by activating Nrf2 (Katsumata et al., 2014). Human genes, stable agonists and antagonists, and physiological functions mediated by DP, CRTH2, and FP receptors are also shown in Supplementary Table 3. The KO mice of *Ptgdr* (for DP, Matsuoka et al., 2000), *Ptgdr2* (for CRTH2, Satoh et al., 2006) and *Ptgfr* (FP, Sugimoto et al., 1997) genes and the flox mice of *Ptgdr* (Kong et al., 2016) and *Ptgfr* (Wang et al., 2018) genes are already generated. The flox mice of *Ptgdr2* gene are not yet available.

The pathophysiological function of PGD_2_ was extensively studied in the last two decades by pharmacological analyses with selective inhibitors for L-PGDS and H-PGDS, agonists and antagonists for DP, CRTH2 and FP receptors, as well as by *in vivo* analyses with various gene-manipulated mice of *L-Pgds*, *HPgds*, *Ptgdr*, *Ptgdr2* and *Ptgfr* genes, as described in the later sections.

The cDNA and genome cloning of *L-Pgds* (Nagata et al., 1991; Igarashi et al., 1992) revealed that L-PGDS is a member of the lipocalin family, as judged by the homology of amino acid sequence and gene structure. The gene structure of *L-Pgds* (Igarashi et al., 1992) is remarkably analogous to those of other lipocalins, such as β-lactoglobulin, α2-urinary globulin, placental protein 14, and α1-microglobulin. All those proteins have the same numbers and sizes of exons and phase of splicing of introns. Positions of exon/intron junction of the *L-Pgds* gene are highly conserved and located around the same positions of those other lipocalins, in a multiple alignment of amino acid sequences despite a weak homology (Urade and Hayaishi, 2000a; Urade et al., 2006).

In 1993, the amino acid sequence of β-trace protein purified from human cerebrospinal fluid (CSF, Hoffmann et al., 1993) was found to be exactly identical to that of human L-PGDS (Nagata et al., 1991) after cleavage of its N-terminal hydrophobic signal sequence. β-Trace is a major protein of human CSF which was originally found in human CSF in 1961 (Clausen, 1961), but its molecular properties and physiological functions remained unidentified. In 1994, we purified L-PGDS from human CSF and confirmed that CSF L-PGDS is enzymatically and immunologically the same as β-trace (Watanabe et al., 1994).

Lipocalin-type prostaglandin D_2_ synthase is distributed in the CNS (Urade et al., 1989, 1993), male genital organs (Urade et al., 1989; Tokugawa et al., 1998) and human heart (Eguchi et al., 1997), and secreted into CSF (Hoffmann et al., 1993; Watanabe et al., 1994), seminal plasma (Tokugawa et al., 1998) and plasma during coronary circulation (Eguchi et al., 1997), respectively. L-PGDS maintains the binding activity of lipophilic ligands similar to other members of lipocalin superfamily (Urade et al., 2006), suggesting that L-PGDS acts as an extracellular transporter of lipophilic substances, as described below.

## Ligand Binding Properties of Lipocalin-Type Prostaglandin D_2_ Synthase as an Extracellular Transporter

Supplementary Table 4 summarizes various types of ligands bound to L-PGDS and their binding affinities and kinetics. L-PGDS binds retinoids (Tanaka et al., 1997), thyroids (Beuckmann et al., 1999), and various types of lipophilic ligands. L-PGDS binds also amyloid β (Aβ) peptides (Kanekiyo et al., 2007), prevents the formation of amyloid fibrils (Kanekiyo et al., 2007; Kannaian et al., 2019) and disaggregates the fibrils (Kannaian et al., 2019), acting as a major Aβ chaperone in human CSF. L-PGDS binds PGD_2_ at a molar ratio of 1:2 with high and low affinities, suggesting that L-PGDS may function as an extracellular PGD_2_-transporter in the absence of substrate PGH_2_ (Shimamoto et al., 2021). All those ligands exhibit binding affinities to L-PGDS much higher than the Km value of PGH_2_ but do not potently inhibit the L-PGDS activity. L-PGDS has a large central hydrophobic cavity within a molecule, in which two to three molecules of those small ligands are captured. Docking analyses suggest that those ligands bind to the hydrophobic pocket at the bottom of a large central cavity of L-PGDS, which is different from the PGH_2_-binding catalytic pocket at the upper entrance of L-PGDS, as described later.

### Binding of Lipophilic Hormones Including Retinoids and Thyroids

Lipocalin-type prostaglandin D_2_ synthase binds *all-trans*- and *9-cis-*retinoic acids and *all-trans-* and 13-*cis*-retinal, but not retinol, with high affinities of Kd = 70–80 nM at a 1:1 molar ratio (Tanaka et al., 1997; Shimamoto et al., 2007; Inoue et al., 2009), similar to several other lipocalins. L-PGDS/β-trace is secreted into various body fluids, such as CSF of the brain (Hoffmann et al., 1993; Watanabe et al., 1994), interphotoreceptor matrix of the retina (Beuckmann et al., 1996), plasma (Eguchi et al., 1997), and seminal plasma (Tokugawa et al., 1998), suggesting that L-PGDS acts as an extracellular transporter of those retinoids within these compartments. Retinoid transporting function by L-PGDS is demonstrated in the study of glial migration (Lee et al., 2012) and the placode formation in *Xenopus* embryo (Jaurena et al., 2015) by using the Cys65Ala mutant without the PGD_2_ synthase activity (Urade et al., 1995).

Thyroid hormones, such as thyroxine (T4), 3-3′,5′-triiodo-L-thyronine (T3) and 3-3′,5-triiodo-L-thyronine (reverse T3), bind to L-PGDS with affinities of Kd = 0.7 to 3 μM (Beuckmann et al., 1999). L-PGDS expression is upregulated in rat brain through activation of a thyroid hormone/retinoic acid-responsive element in the promoter region of the rat *L-Pgds* gene (García-Fernández et al., 1998). Thyroid hormones upregulate *L-Pgds* gene expression in the male genital organs, the seminal vesicle and testis, of cat fish (Sreenivasulu et al., 2013). These results suggest that L-PGDS gene expression is controlled by its own ligands by an autonomic positive regulation mechanism. Fish L-PGDS ortholog does not contain an active thiol of Cys65 in mammalian L-PGDSs so that the fish L-PGDS does not show the PGD_2_ synthase activity but maintains the retinoid/thyroid-binding activity (Fujimori et al., 2006). Therefore, the fish L-PGDS is also predicted to function as a non-enzymic transporter of lipophilic hormones (Sreenivasulu et al., 2013).

### Binding of Heme-Degradation Products and Heme

Lipocalin-type prostaglandin D_2_ synthase binds bilirubin and biliverdin (Beuckmann et al., 1999; Inoue et al., 2009; Miyamoto et al., 2009; Sreenivasulu et al., 2013), harmful degradation products of heme with high affinities (Kd of 20–40 nM) (Tanaka et al., 1997). This binding is also confirmed in the CSF of patients after subarachnoid hemorrhage, in which a part of biliverdin is covalently bound to the Cys65 residue, an active thiol of L-PGDS, as demonstrated by NMR (Inui et al., 2014). Therefore, L-PGDS scavenges those harmful heme-metabolites from CSF. L-PGDS also binds heme itself, as examined by NMR (Phillips et al., 2020). The heme-binding L-PGDS is associated with the pseudo-peroxidase activity, which is proposed to contribute to the anti-apoptotic activity of L-PGDS against the H_2_O_2_-induced cytotoxicity (Phillips et al., 2020).

### Binding of Amyloid β Peptides

Lipocalin-type prostaglandin D_2_ synthase binds to Aβ peptides 1–40 and 1–42, and their fibrils with high affinities (Kd = 18–50 nM). The aggregation of Aβ peptides is crucial in the pathogenesis of Alzheimer’s disease. L-PGDS recognizes a region of amino acid residues of 25–28 in Aβ peptides, the key region for conformational change to β-sheet structures (Kanekiyo et al., 2007). L-PGDS inhibits the spontaneous aggregation of Aβ (1–40) and Aβ (1–42) in a physiological concentration range from 1 to 5 μM in human CSF, and also prevents the seed-dependent aggregation of 50 μM Aβ (1–40) with Ki of 0.75 μM. When L-PGDS is removed from the human CSF by immunoaffinity chromatography with anti-(L-PGDS) IgG, the inhibitory activity toward Aβ (1–40) aggregation in human CSF decreases by 60%. Recombinant human L-PGDS disaggregates Aβ fibrils and dissolves many insoluble proteins existed in amyloid plaques in the brain of patients with Alzheimer’s disease (Kannaian et al., 2019). These results indicate that L-PGDS is a major endogenous Aβ-chaperone in the human brain.

### Binding of Lipids, Gangliosides, and Lipophilic Drugs

Lipocalin-type prostaglandin D_2_ synthase also binds gangliosides, such as GM_1_ and GM_2_, with a high affinity with Kd = 65–210 nM (Mohri et al., 2006a). L-PGDS is upregulated in oligodendrocytes and a few neurons in the brain of murine models of various lysosomal storage diseases, such as Krabbe’s disease (Taniike et al., 1999; Mohri et al., 2006b), Tay–Sachs disease, Sandhoff disease, GM_1_ gangliosidosis and Niemann–Pick type C1 disease (Mohri et al., 2006a). These results suggest that L-PGDS plays a protective role on oligodendrocytes in scavenging harmful lipophilic substrates accumulated by malfunction of myelin metabolism in lysosomal storage diseases, as demonstrated by using double mutant mice with *L-Pgds* gene KO mice as shown later (Taniike et al., 2002).

Lipocalin-type prostaglandin D_2_ synthase also binds fatty acids (Zhou et al., 2010; Elmes et al., 2018) and various water insoluble drugs, including a sleeping pill Diazepam (Fukuhara et al., 2012a), a drug used for high blood pressure Telmisartan (Mizoguchi et al., 2015), anti-cancer drugs (Nakatsuji et al., 2015; Teraoka et al., 2017), cannabinoid receptor antagonists (Yeh et al., 2019), cannabinoid metabolites (Elmes et al., 2018), synthetic cannabinoids (Elmes et al., 2018), and anti-cholinergic drugs (Low et al., 2020), as listed in Supplementary Table 4. The binding affinities for those compounds are lower than those for retinoids, thyroids and Aβ. The drug-binding ability of L-PGDS is used for the drug delivery system and is recognize to induce off-target effects of anti-cholinergic drugs, such as Chlorpheniramine and Trazodone, to modulate the cytotoxicity of Aβ fibrils (Low et al., 2020).

### Binding of Nicotinamide Coenzymes

Lipocalin-type prostaglandin D_2_ synthase binds NADPH, NADP^+^, and NADH, as examined by thermodynamic and NMR analyses. These hydrophilic ligands, especially NADPH, interact with the upper pocket of a ligand-binding cavity of L-PGDS with an unusual bifurcated shape (Qin et al., 2015). The binding affinity of L-PGDS for NADPH is comparable to that of NADPH oxidases. Therefore, L-PGDS may attenuate the NADPH oxidase activities through interaction with NADPH, being involved in anti-oxidative stress function of L-PGDS.

### Binding of Substrate Analog and Product

Most recently, we demonstrated by isothermal titration assay and NMR analyses that L-PGDS binds a stable PGH_2_ analog U-46619 at two binding sites of high and low affinities with Kd values of 0.53 and 7.91 μM, respectively, and also its product PGD_2_ with 0.3 and 44 μM, respectively, in the hydrophilic catalytic pocket at the upper entrance of L-PGDS molecule (Shimamoto et al., 2021). The high affinity binding is lost in the Cys65Ala mutant of L-PGDS, indicating that the thiol group of Cys65 is important for high affinity binding of the substrate PGH_2_ and the product PGD_2_ to L-PGDS. On the other hand, the low affinity binding site has a wide binding spectrum for other PGs including PGE_2_ and PGF_2__α_ with comparable affinities. These results indicate the substrate-induced catalytic mechanism for L-PGDS. The sleep-inducing activity by an intracerebroventricular infusion of PGD_2_ is significantly reduced in *L-Pgds* gene KO mice than wild-type mice, suggesting that L-PGDS binds and transport PGD_2_ in the brain to stimulate effectively DP receptors in the sleep-promoting system (Shimamoto et al., 2021).

## Structural Characterization of Lipocalin-Type Prostaglandin D_2_ Synthase by Nuclear Magnetic Resonance and X-Ray Crystallography

**FIGURE 2:**
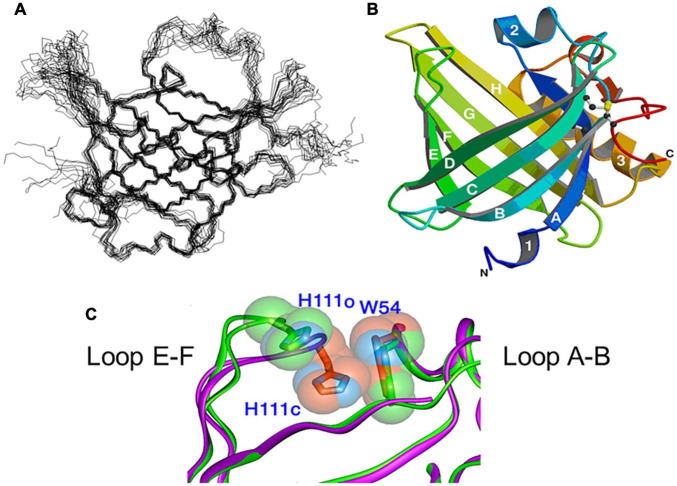
NMR **(A)** and X-ray crystallographic **(B)** structures of L-PGDS (Shimamoto et al., 2007; Kumasaka et al., 2009, respectively). **(C)** Overlapping views of the entrance of the catalytic pocket of the open (*green*) and closed (*purple*) conformers of L-PGDS (Kumasaka et al., 2009). Positions of EF-loop and H2-helix are indicated.

**FIGURE 3:**
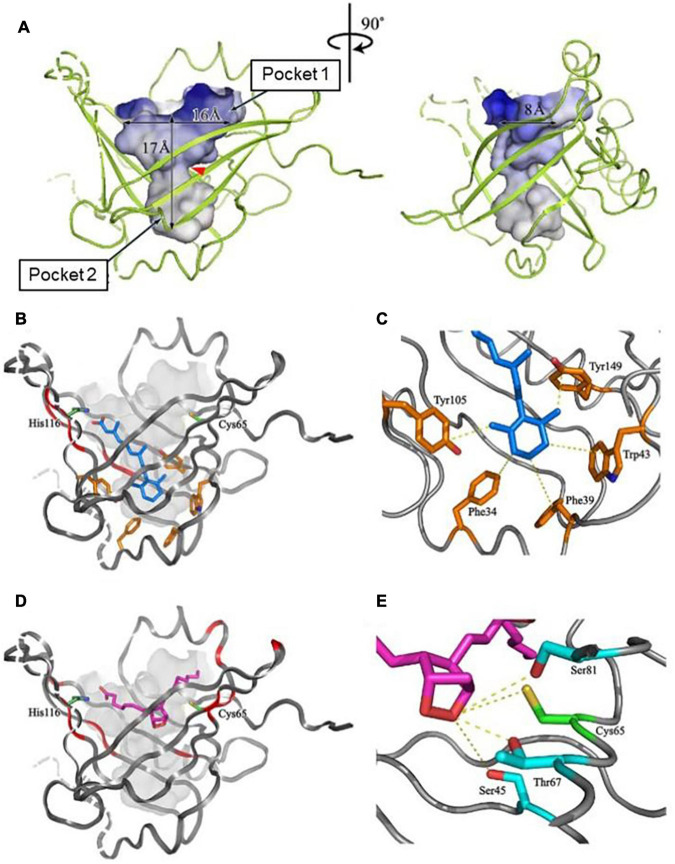
The structure of a large central cavity of L-PGDS **(A)** and docking models of the complexes with *all-trans*-retinoic acid **(B,C)** or PGH_2_ **(D,E)** determined by NMR (Shimamoto et al., 2007). The molecule of *all-trans*-retinoic acid are shown in *light blue* **(B,C)**. The carbon chain of PGH_2_ is shown in *purple* and a 9,11-endoperoxide group, in *orange* **(D,E)**. Amino acid residues important for the ligand binding and the catalytic activity are shown in panels **(C,E)**, respectively.

Figure 2 shows NMR (Figure 2A) and X-ray crystallographic structures (Figures 2B,C) of recombinant L-PGDS expressed in *E. coli*. We first determined in 2007 the solution structure of mouse L-PGDS by NMR (Shimamoto et al., 2007). L-PGDS possesses a typical lipocalin fold of β-barrel with two sets of β-sheet composed of each four strands of anti-parallel β-sheet and a 3-turn α-helix associated with the outer surface of the barrel. L-PGDS possesses a large central cavity with a flexible rid of a wide entrance opening to the upper end of the barrel. The central cavity of L-PGDS is larger than those of other lipocalins and contains two pockets (Figure 3A). NMR titration analyses demonstrate that *all-trans-*retinoic acid occupies the hydrophobic pocket 2 with amino acid residues important for the retinoid binding well conserved in other lipocalins (Figures 3B,C) and that PGH_2_ occupies the hydrophilic pocket 1 containing Cys65 and a hydrogen network of Ser45, Thr67, and Ser81 (Figures 3D,E).

**FIGURE 4:**
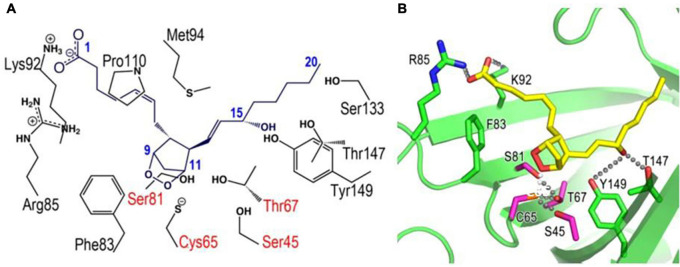
Docking models of L-PGDS complexes with PGH_2_ based on X-ray crystallography (Kumasaka et al., 2009). Amino acid residues important for interaction with PGH_2_ are shown in panel **(A)**. R85, K92, F83, S82, C65, S45, T67, Y149, and T147 are shown in stick-model form in panel **(B)**. Hydrogen bonding network around Cys65 and 15-hydroxy group of PGH_2_ are also indicated in panel **(B)**.

We determined in 2009 the crystal structures of mouse L-PGDS Cys65Ala mutant (Kumasaka et al., 2009) and revealed that L-PGDS exhibits two different conformers due to the movement of the flexible EF-loop (Figures 2A,C). One conformer of L-PGDS has an open cavity of the β-barrel and the other, a closed cavity (Figure 2C). The upper hydrophilic pocket 1 of the central cavity contains the catalytically essential Cys65 residue with a hydrogen bond network with Ser45, Thr67, and Ser81 (Figures 3E, 4). The SH titration analyses combined with site-directed mutagenesis (Kumasaka et al., 2009) demonstrate that Cys65 residue is activated by its interaction with Ser45 and Thr67. The crystal structure of L-PGDS confirmed that the lower compartment of the central cavity is composed of hydrophobic amino acid residues highly conserved among other lipocalins (Figure 3C).

X-ray crystallographic structures of human L-PGDS was also reported, in which fatty acids (Zhou et al., 2010) and polyethylene glycol used as precipitants (Perduca et al., 2014) are identified to be inserted into the central cavity of the molecule. The NMR structures of L-PGDS complexed with an L-PGDS-selective inhibitor AT56 (Irikura et al., 2009) or a variety of lipophilic (Qin et al., 2015) or hydrophobic (Shimamoto et al., 2007; Sreenivasulu et al., 2013; Inui et al., 2014; Kannaian et al., 2019; Low et al., 2020; Phillips et al., 2020) ligands were also already reported (Supplementary Table 4). Those structural information is useful for designing inhibitors specific for L-PGDS.

**FIGURE 5:**
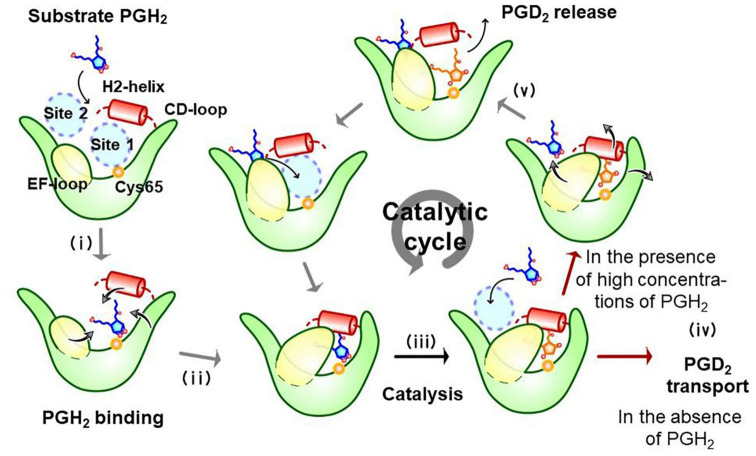
Substrate-induced product-release mechanism for L-PGDS (Shimamoto et al., 2021).

Figure 5 shows a substrate-induced product release mechanism for L-PGDS (Shimamoto et al., 2021). L-PGDS has two binding sites for the substrate PGH_2_ and the product PGD_2_ in the pocket 1 of the upper part of the cavity. Site 1 is the catalytic site containing Cys65 and site 2, the non-catalytic site. Apo-form of L-PGDS has a wide open entrance, through which PGH_2_ enters to the pocket 1. PGH_2_ binds to the site 1 at step (i). H2-helix, CD-loop, and EF-loop of L-PGDS interact with PGH_2_ so that the cavity is closed at step (ii). The closed conformation of L-PGDS holds the 9,11-endoperoxide group of PGH_2_ to interact with the thiol group of the catalytic Cys65. The catalytic reaction occurs to produce PGD_2_ at step (iii). After the catalytic reaction, PGD_2_ still binds to site 1 at the high affinity with Kd of 0.3 μM (Supplementary Table 4). In the absence of PGH_2_, L-PGDS acts as a carrier of PGD_2_ as shown at step (iv). In the presence of excess concentrations of PGH_2_, the next molecule of PGH_2_ binds to site 2, inducing movement of CD-loop to open site 1 and release PGD_2_ at step (v). PGH_2_ then move from site 2 to site 1 and the catalytic cycle starts again (Shimamoto et al., 2021).

Post transcriptional modification of L-PGDS/β-trace in human CSF is identified by a proteoform analysis (Zhang et al., 2014) to be *N*-glycans at Asn51 and Asn78 with different *N*-glycan compositional variants, a core-1 HexHexNAc-O-glycan at Ser29, acetylation at Lys38 and Lys160, sulfonation at Ser63 and Thr164, and dioxidation at Cys65 and Cys167 after cleavage of an N-terminal signal sequence to Ala22. Two N-truncated forms of L-PGDS from Gln31 and Phe34 exist in human urine (Nagata et al., 2009).

A loop structure of residues 55–63 between β-strand A and B of L-PGDS is proposed to be homologous to a sequence domain in apolipoprotein E responsible for binding to low-density lipoprotein (LDL) receptor (Portioli et al., 2017). Two potential GTPase Rab4-binding sites are proposed to locate residues 75–98 and 85–92 within β-strand B and C of human L-PGDS (Binda et al., 2019).

## Cell Culture Studies of Lipocalin-Type Prostaglandin D_2_ Synthase

Lipocalin-type prostaglandin D_2_ synthase is constitutively expressed in several cell lines including human brain-derived TE671 cells, human neuroblastoma SH-SY5Y cells, mouse adipocytic 3T3-L1 cells, and others. Several types of primary cultured cells, such as leptomeningeal cells, vascular endothelial cells, bone marrow-derived macrophages or mast cells are also used to study the regulation mechanism of *L-Pgds* gene and the functional analyses of L-PGDS, as summarized in Supplementary Table 5.

### *L-Pgds* Gene Regulation in the Central Neuronal Cells

In mouse neuronal GT1-7 cells, L-PGDS is induced by dexamethasone (García-Fernández et al., 2000). The L-PGDS induction is suppressed by 12-*O*-tetradecanoyl phorbol 13-acetate (TPA), whereas TPA induces the synthesis of PGs in many tissues. The L-PGDS induction by glucocorticoids is also found in mouse adipocytic 3T3-L1 cells (Yeh et al., 2019). Dexamethasone and glucocorticoids are known to suppress inflammation by inhibition of PG production. However, PGD_2_ produced by L-PGDS may, in part, be also involved anti-inflammatory effects by those hormones.

In rat leptomeningeal cells, the Notch-Hes signal represses *L-Pgds* gene expression by interaction with an atypical E-box (aE-box) in the promoter region. IL-1β upregulates *L-Pgds* gene expression through the NF-kB pathway at two NF-kB sites of the *L-Pgds* gene (Fujimori et al., 2003) and by contact with astrocytes (Fujimori et al., 2007b).

**FIGURE 6:**
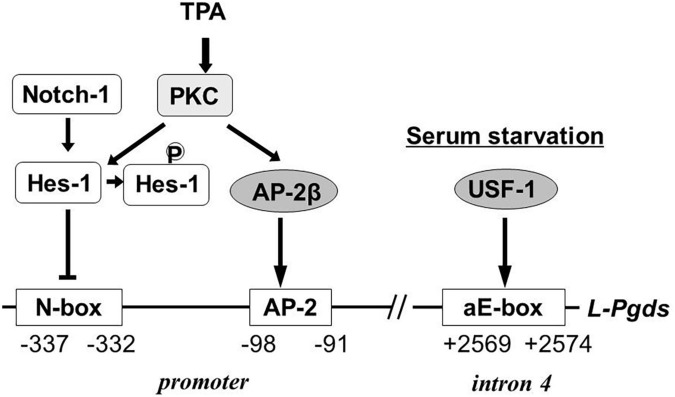
Regulatory mechanism of human *L-Pgds* gene expression in brain-derived TE671 cells (Fujimori et al., 2005, 2008; Fujimori and Urade, 2007). *L-Pgds* gene expression is lowered by the Notch-Hes signal via an N-box, but enhanced by AP-2β through the AP-2 element. TPA activates PKC, followed by phosphorylation of Hes-1 to cancel the binding of Hes-1 to the N-box. Upstream stimulatory factor-1 (USF-1) activates *L-Pgds* gene expression through the atypical E-box (aE-box) in the intron 4. Serum starvation induces further *L-Pgds* gene expression.

In human TE671 cells, the Notch-Hes signal represses *L-Pgds* gene expression by interaction with an N-box in the promoter region and the AP-2β binding to the AP-2 element in the promoter region is involved in maintenance of *L-Pgds* gene expression (Fujimori et al., 2005). TPA induces L-PGDS in TE671 cells. Protein kinase C phosphorylates Hes-1, inhibits DNA binding of Hes-1 to the N-box, and induce L-PGDS. Activation of the AP-2β function is involved in up-regulation of *L-Pgds* gene expression. The aE-box is critical for transactivation of the *L-Pgds* gene in TE671 cells. Upstream stimulatory factor (USF)-1 binds to the aE-box in intron 4 of the human *L-Pgds* gene and activates *L-Pgds* gene expression. Binding of AP-2β in the promoter also cooperatively contributes to the transactivation of *L-Pgds* gene (Fujimori and Urade, 2007). Serum starvation induces PGD_2_ production in TE671 cells through transcriptional activation of *Pghs-2* and *L-Pgds* genes. The serum starvation up-regulates *L-Pgds* gene expression by binding of USF-1 to aE-box within intron 4. The USF1 expression is enhanced through activation of p38 mitogen-activated protein kinase in TE671 cells (Fujimori et al., 2008). Figure 6 summarizes the transcriptional regulation of the human *L-Pgds* gene in TE671 cells.

In human neuroblastoma SH-SY5Y cells, L-PGDS prevents neuronal cell death caused by oxidative stress (Fukuhara et al., 2012b). An NF-kB element in the proximal promoter region of *L-Pgds* gene mediates paraquat-induced apoptosis of these cells (Fujimori et al., 2012a).

In U251 glioma cell line expressing estrogen receptor (ER) α, estradiol (10^–11^ M) increases the promoter activity of *L-Pgds* gene. Conditioned media from estradiol-treated neurons increases the *L-Pgds* gene promoter activity in glial cells, suggesting that a paracrine factor released from the neighboring neurons after stimulation of estrogen induces L-PGDS in glial cells (Devidze et al., 2010).

In primary cultured astrocytes, microglial cells, and fibroblasts (Lee et al., 2012), L-PGDS accelerates the migration of these cells and changes the morphology to the characteristic phenotype in reactive gliosis. Activation of AKT, RhoA, and JNK pathways mediates L-PGDS-induced cell migration. L-PGDS interacts with myristoylated alanine-rich protein kinase C substrate (MARCKS) and promotes the cell migration in a PGD_2_-independent manner, because that the inactive Cys65Ala mutant without the PGD_2_ synthase activity shows the same effect.

### *L-Pgds* Gene Regulation in Vascular Cells

Fluid shear stress induces L-PGDS in human vascular endothelial cells (Taba et al., 2000). c-Fos and c-Jun bind to the AP-1 binding site of the 5′-promoter region of the *L-Pgds* gene to induce L-PGDS. Shear stress elevates the c-Jun phosphorylation level in a time-dependent manner, similar to that of *L-Pgds* gene expression. A c-Jun N-terminal kinase inhibitor decreases the c-Jun phosphorylation, DNA binding of AP-1, and shear stress-induced *L-Pgds* gene expression (Miyagi et al., 2005). Homozygosity for the C variant of the T-786C single-nucleotide polymorphism of the human endothelial NO synthase gene exhibits a reduced endothelial cell capacity to generate NO and is less sensitive to fluid shear stress. In the CC-genotype of endothelial cells, fluid shear stress elicits a marked rise in *Pghs-2* and *L-Pgds* gene expression (Urban et al., 2019). These results suggest that the increased production of PGD_2_ is the reason why CC-genotype endothelial cells maintain the robust anti-inflammatory characteristics, despite a reduced capacity to produce NO.

Exogenously added L-PGDS induces apoptosis of epithelial, neuronal and vascular smooth muscle cells. L-PGDS-induced apoptosis is inhibited by mutations in a glycosylation site Asn51, a putative protein kinase C phosphorylation site Ser106, and the enzymatic active site Cys65, although the action mechanism remains unclear (Ragolia et al., 2007).

### *L-Pgds* Gene Regulation in Adipocytes

**FIGURE 7:**
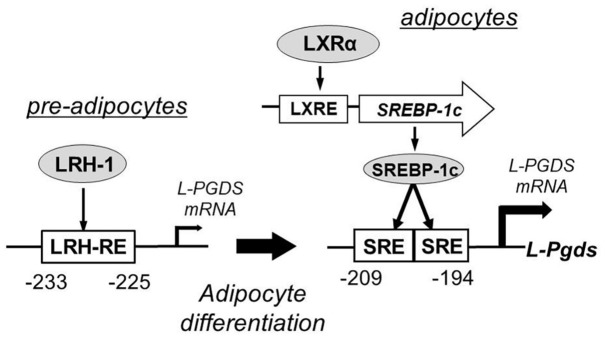
Transcriptional regulation of mouse *L-Pgds* gene in adipocyte 3T3-L1 cells (Fujimori et al., 2007a). *L-Pgds* gene expression is activated by liver receptor homolog-1 (LRH-1), one of orphan nuclear receptors, through the LRH-responsive element (LRH-RE) in pre-adipocytes. In the differentiated adipocytes, activation of liver X receptor (LXR) elevates the expression of sterol regulatory element-binding protein-1 (SREBP-1c) through the LXR-responsive element (LXRE), and then SREBP-1c strongly enhances the *L-Pgds* gene expression via two sterol regulatory elements (SREs) in the promoter region of *L-Pgds* gene.

Lipocalin-type prostaglandin D_2_ synthase is involved in adipocyte differentiation of mouse 3T3-L1 cells (Fujimori et al., 2007a). A responsive element for liver receptor homolog-1 (LRH-1) in the promoter region of the *L-Pgds* gene plays a critical role in *L-Pgds* gene expression in pre-adipocytes of 3T3-L1 cells. Two sterol regulatory elements (SREs) in the promoter region act as *cis*-elements for activation of *L-Pgds* gene. Synthetic liver X receptor agonist T0901317 activates the expression of SRE-binding protein-1c (SREBP-1c) and upregulates *L-Pgds* gene expression in these cells. LRH-1 and SREBP-1c bind to their respective binding elements in the promoter of *L-Pgds* gene and increase *L-Pgds* gene expression in pre-adipocytes and adipocytes, respectively, of 3T3-L1 cells. Figure 7 summarizes the transcriptional regulation of the mouse *L-Pgds* gene in preadipocytes and adipocytes of mouse 3T3-L1 cells.

Gene knockdown of *L-Pgds* by antisense *L-Pgds* gene in pre-adipocytes stimulates fat storage during the maturation stage of these cells (Chowdhury et al., 2011). In these cells, Δ^12^- prostaglandin J_2_ (Δ^12^- PGJ_2_), a dehydrated product of PGD_2_ produced by L-PGDS, activates adipogenesis through both PPARγ-dependent and -independent pathways (Fujimori et al., 2012b), although the J series of PGD_2_ is not physiologically produced *in vivo* as described below.

Prostaglandin D_2_ is relatively unstable in water, as compared with PGE_2_ and PGF_2__α_, both of which are chemically very stable for several months to years. PGD_2_ is spontaneously dehydrated in water to prostaglandin J_2_ (PGJ_2_), Δ^12^-PGJ_2_ and 15-deoxy-Δ^12,14^-PGJ_2_ (15d-PGJ_2_). These cyclopentene PGs with a O=C-C=C- bond are chemically reactive with various SH and amino groups to make conjugates. As d15-PGJ_2_ acts as a ligand of PPARγ *in vitro*, several reports emphasize the importance of d15-PGD_2_ *in vivo*. However, those cyclopentene PGs are not produced physiologically *in vivo* and are artificially generated from PGD_2_ during extraction and purification for measurement. Those cyclopentene PGs are cytotoxic and induce apoptosis of cultured cells because of their massive reactivities. We have to be careful about the artificial cytotoxic effect of those dehydrated PGD_2_ products in the cell culture system. In cases that the target receptor of PGD_2_ is predicted, it is desirable to use chemically stable agonists, rather than PGD_2_ itself.

Adipocytes dominantly express CRTH2 receptors (Wakai et al., 2017). CRTH2 antagonist, but not DP antagonist, suppresses the PGD_2_-elevated intracellular triglyceride level. CRTH2 agonist 15R-15-methyl PGD_2_ increases the mRNA levels of the adipogenic and lipogenic genes, decreases the glycerol release level, and represses the forskolin-mediated increase of cAMP-dependent protein kinase A activity (PKA) and phosphorylation of hormone-sensitive lipase (HSL). The lipolysis is enhanced in the adipocytes differentiated from embryonic fibroblasts of CRTH2 KO mice. These results indicate that PGD_2_ produced by L-PGDS suppresses the lipolysis by repression of the cAMP-PKA-HSL axis through CRTH2 receptors in adipocytes in an autocrine manner.

Glucocorticoid induces *L-Pgds* gene expression and leptin production in differentiated primary preadipocytes (Yeh et al., 2019). *L-Pgds* siRNA and L-PGDS inhibitor AT56 suppress glucocorticoid-induced leptin production, whereas overexpression of *L-Pgds* gene enhances leptin production. The L-PGDS-leptin pathway may be involved in undesired effects of clinical used glucocorticoid including obesity. In human mesenchymal stroma cells used in cellular therapies, L-PGDS is involved in differentiation of adipocytes, suggesting that *L-Pgds* gene expression is a potential quality marker for these cells, as it might predict unwanted adipogenic differentiation after the transplantation (Lange et al., 2012).

### Lipocalin-Type Prostaglandin D_2_ Synthase Studies in Skeletal Muscle Cells

Lipocalin-type prostaglandin D_2_ synthase stimulates glucose transport of the insulin-sensitive rat skeletal muscle cell line L6 at the basal level and after insulin-stimulation. L-PGDS increases translocation of glucose transporter 4 to the plasma membrane, suggesting that L-PGDS, via production of PGD_2_, is an important mediator of muscle and adipose glucose transport, which plays a significant role in the glucose intolerance associated with type 2 diabetes (Ragolia et al., 2008).

### *L-Pgds* Gene Regulation in Peripheral Nervous Cells

*L-Pgds* gene is the most upregulated gene in the primary culture of rat dorsal root ganglion (DRG) neurons infected with the intracellular domain of neuregulin 1 type III, a member of the neuregulin family of growth factors. DRG neurons secrete L-PGDS and PGD_2_, the latter of which stimulates CRTH2 receptors and enhances myelination of Schwann cells (Trimarco et al., 2014).

Lipopolysaccharide (LPS) stimulation increases *L-Pgds* gene expression in intestinal neurons and glial cells in the primary culture of rat enteric nervous system. L-PGDS inhibitor AT56 inhibits PGD_2_ production in the primary culture treated with LPS. As *Pghs-2* and *L-Pgds* gene expression increase in the inflamed colonic mucosa of patients with active Crohn’s disease, the L-PGDS pathway may be a new therapeutic target in this disease (Le Loupp et al., 2015).

### *L-Pgds* Gene Regulation in Macrophages

Incubation with LPS or *Pseudomonas* upregulates *L-Pgds* gene expression in mouse bone marrow-derived macrophages and macrophage cell line RAW 264.7 cells, in which AP-1 and p53 regulate the *L-Pgds* gene expression positively and negatively, respectively (Joo et al., 2007). Binding of PU.1, a transcription factor essential for macrophage development and inflammatory gene expression, to the cognate site in the *L-Pgds* gene promoter mediates the L-PGDS induction. LPS stimulation triggers TLR4 signaling and activates casein kinase II (CKII). CKII phosphorylates PU.1 at Ser148. The activated phosphorylated PU.1 binds to its cognate site in the promoter. The TLR4 signaling also activates JNK and p38 kinase that phosphorylate and activate cJun. Activated phosphorylated cJun binds to both PU.1 and the AP-1 site of the promoter. PU.1 and cJun form a transcriptionally active complex in the *L-Pgds* gene promoter, leading to *L-Pgds* gene expression (Joo et al., 2009). Therefore, L-PGDS is also important for the innate immunity.

### *L-Pgds* Gene Regulation in Chondrocytes

*L-Pgds* gene expression is upregulated in human primary cultured chondrocytes by treatment with IL-1β. Inhibitors of MAPK p38, c-jun N-terminal kinase (JNK), and the NF-κB/Notch signaling pathways suppress the *L-Pgds* gene upregulation (Zayed et al., 2008).

### Lipocalin-Type Prostaglandin D_2_ Synthase Studies in Mast Cell Maturation

Lipocalin-type prostaglandin D_2_ synthase in fibroblasts is important for maturation of mouse and human mast cells (Taketomi et al., 2013). Mast cells secrete a group III phospholipase A_2_ (PLA2G3), a mammalian ortholog of anaphylactic bee venom phospholipase A_2_. The secreted PLA2G3 couples with L-PGDS in neighboring fibroblasts to provide PGD_2_. The PGD_2_ produced by fibroblasts stimulates DP receptors on mast cells to facilitate maturation of mast cells. Mast cells maturation and anaphylaxis are impaired in KO mice of PLA2G3, L-PGDS or DP, mast cell–deficient mice reconstituted with PLA2G3-null or DP-null mast cells, or mast cells cultured with *L-Pgds* gene–ablated fibroblasts. The PLA2G3-L-PGDS-DP paracrine axis is important for the innate immunity and inflammation by the mast cell-fibroblast interaction.

### Lipocalin-Type Prostaglandin D_2_ Synthase Studies in Cells of the Skin

Follicular melanocytes in the mouse skin express L-PGDS. B16 mouse melanoma cells express L-PGDS under the control of microphthalmia-associated transcription factor (MITF) responsible for differentiation of melanocytes (Takeda et al., 2006). In human epidermal keratinocytes, antimycotics induce PGD_2_ release and suppress the expression of thymic stromal lymphopoietin, the NF-kB activity, and IkBα degradation induced by poly I:C plus IL-4. L-PGDS inhibitor AT-56 counteracts the antimycotic-induced suppression of thymic stromal lymphopoietin production and the NF-kB activity (Hau et al., 2013).

### Lipocalin-Type Prostaglandin D_2_ Synthase Studies in Cancer Cells

Overexpression of Yes-associated protein 1 (YAP) suppresses *L-Pgds* and *Ptgdr2* gene expression in gastric cancer cells. Overexpression of *L-Pgds* and *Ptgdr2* genes decreases proliferation and self-renewal of gastric cancer cells by YAP. These results indicates that YAP inhibits *L-Pgds* and *Ptgdr2* gene expression to promote self-renewal of gastric cancer cells (Bie et al., 2020). Chemotherapeutics induce Cox-2 and lead apoptosis of human cervical carcinoma cells. *L-Pgds* gene knockdown by siRNA prevents the chemotherapeutics-induced apoptosis, suggesting that the apoptosis occurs through PGD_2_ production by L-PGDS (Eichele et al., 2008).

### Lipocalin-Type Prostaglandin D_2_ Synthase Studies in Cells of the Prostate Gland

Bisphenol A, a synthetic plasticizer widely used to package daily necessities, induces *Pghs2* and *L-Pgds* gene expression in human prostate fibroblasts and epithelial cells. Cox-2 inhibitor NS398 and L-PGDS inhibitor AT56 suppress the cell proliferation enhanced by bisphenol A and increase apoptosis of those cells. Thus, Cox-2 and L-PGDS mediate low-dose bisphenol A-induced prostatic hyperplasia through pathways involved in cell proliferation and apoptosis (Wu et al., 2020).

### Lipocalin-Type Prostaglandin D_2_ Synthase Studies in Seminal Plasma and Oviduct Fluid

The seminal plasma and oviduct fluid contain L-PGDS. Pretreatment of bovine sperm and/or oocytes with anti-L-PGDS antibody inhibits *in vitro* fertilization and increases sperm-oocyte binding (Gonçalves et al., 2008a). Anti-L-PGDS antibody reacts with cow oocytes incubated with oviduct fluid. Pretreatment of oocytes with anti-L-PGDS antibody inhibits sperm binding, fertilization and embryonic development *in vitro* (Gonçalves et al., 2008b).

### Amphibian Ortholog of Lipocalin-Type Prostaglandin D_2_ Synthase and Its Function During Development of *Xenopus* Embryo

Amphibian ortholog of *L-Pgds* gene is identified and its protein is expressed in *Xenopus* A6 cells. Amphibian ortholog of L-PGDS is associated with both PGD_2_ synthase activity and *all-trans-*retinoic acid-binding activity (Irikura et al., 2007). During development of *Xenopus* embryo, amphibian *L-Pgds* gene expression is induced by zinc-finger transcription factor Zic 1 specifically at the anterior neural plate and allows for the localized production and transport of retinoic acid, which in turn activates a cranial placode developmental program in neighboring cells (Jaurena et al., 2015). This effect is reproduced by the Cys65Ala mutant of amphibian L-PGDS, indicating that amphibian L-PGDS functions independently of its enzymatic activity yet as an extracellular retinoic acid transporter.

### Intracellular Interaction of Lipocalin-Type Prostaglandin D_2_ Synthase With D Type of Prostanoid Receptors in HEK293 or HeLa Cells

Lipocalin-type prostaglandin D_2_ synthase interacts intracellularly with DP receptors co-expressed in HEK293 or HeLa cells. L-PGDS or its Cys65Ala mutant promotes cell surface expression of DP receptors, but not of other G-protein coupled receptors including CRTH2 receptors. Interaction of L-PGDS with the C-terminal MEEVD residues of Hsp90 within cells is crucial for export of DP receptors to the cell surface. Co-expression of L-PGDS with DP receptors and Hsp90 promotes PGD_2_ synthesis. Depletion of *L-Pgds* gene decreases DP receptor-mediated ERK1/2 activation. L-PGDS inhibitor AT-56 or DP antagonist BWA868C inhibit L-PGDS-induced ERK1/2 activation. These results indicate that L-PGDS increases the DP receptor-ERK1/2 complex formation and increases DP receptor-mediated ERK1/2 signaling as an intracrine/autocrine signaling mechanism (Binda et al., 2014).

Depletion of endogenous *L-Pgds* gene in HeLa cells decreases recycling of endogenous DP receptors to the cell surface after agonist-induced internalization. *L-Pgds* gene overexpression increases the recycling of DP receptors. Depletion of endogenous GTPase Rab4 prevents L-PGDS-mediated recycling of DP receptors. *L-Pgds* gene depletion inhibits Rab4-dependent recycling of DP receptors. These results indicate that L-PGDS and Rab4 are involved in the recycling of DP receptors. DP receptor stimulation promotes interaction between the intracellular C terminus of DP receptors with Rab4 to form the L-PGDS/Rab4/DP complexes. L-PGDS interacts preferentially with the inactive, GDP-locked Rab4S22N variant rather than with wild-type Rab4 or with constitutively active Rab4Q67L proteins. L-PGDS is involved in Rab4 activation after DP stimulation by enhancing GDP-GTP exchange on Rab4. The region of amino acid residues between 85 and 92 in L-PGDS is proposed to be involved in the interaction with Rab4 and DP recycling, as assessed by deletion mutants and using synthetic peptides (Binda et al., 2019). These mechanisms may amplify the autocrine L-PGDS/PGD_2_/DP receptor function.

## Mammalian Experiments for the Study of Lipocalin-Type Prostaglandin D_2_ Synthase

Lipocalin-type prostaglandin D_2_ synthase is enriched in the CNS, male genital organs, skin of various mammals, and the human heart; and involved in a variety of pathophysiological function. Recent studies using various gene-manipulated mice including systemic or cell/tissue-selective KO mice of *L-Pgds* and *Ptgdr*, *Ptgdr2*, *Ptgfr* genes explore the understanding of the function of L-PGDS, as summarized in Supplementary Table 6.

### Lipocalin-Type Prostaglandin D_2_ Synthase-Mediated Functions in Central Nervous System

In the CNS, L-PGDS is predominantly localized in leptomeningeal cells (arachnoid trabecular cells, arachnoid barrier cells and arachnoid border cells), choroid plexus epithelial cells and oligodendrocytes (Urade et al., 1993; Beuckmann et al., 2000), and is secreted into the CSF to be β-trace (Hoffmann et al., 1993; Watanabe et al., 1994). PGD_2_ is the most potent endogenous sleep-inducing substance (Urade and Hayaishi, 2011) as well as a potent inflammatory mediator (Urade and Hayaishi, 2000b). *L-Pgds* gene KO and TG mice show normal sleep pattern, suggesting that sleep of these mice is normalized by unknown compensation mechanism against gene-manipulation. PGD_2_-induced sleep is mediated by both adenosine A_2A_ receptor-dependent and independent systems (Zhang et al., 2017a). Several cytokines, such as IL-1β and TNFα induce sleep in a PGD_2_-independent manner (Zhang et al., 2017b), Therefore, adenosine and those cytokine systems may be involved in sleep maintenance by compensation for the *L-Pgds* gene deletion. RNA-Seq analyses of the brain of KO mice of *L-Pgds* and/or *ptgdr* genes will provide the information to understand the compensation mechanism.

However, a variety of abnormality was detected in *L-Pgds* gene-manipulated mice after any stimulation or pathological conditions. *L-Pgds* gene KO mice exhibit many abnormalities in the CNS function including regulation of sleep, pain, neural protection, food intake, and others as follows:

#### Pain Regulation

*L-Pgds* gene KO mice do not show allodynia (touch-evoked pain) after an intrathecal administration of PGE_2_- or bicuculline, a GABA_*A*_-antagonist (Eguchi et al., 1999). The PGE_2_- or bicuculline-induced allodynia is reproduced in *L-Pgds* gene KO mice by administration of subfemtomole amount of PGD_2_ (Eguchi et al., 1999). In a rat lumbar disk herniation model with thermomechanical allodynia and degeneration of DRG, overexpression and knockdown of *L-Pgds* gene, respectively, attenuates and worsens the allodynia and tissue degradation (Xu et al., 2021).

#### Sleep Regulation

Human *L-Pgds* gene-overexpressing TG mice show sleep attack with a transient increase in PGD_2_ content in the brain after pain stimulation for tail cutting (Pinzar et al., 2000). *L-Pgds* or *Ptgdr* gene KO mice do not show the rebound sleep after sleep deprivation (Hayaishi et al., 2004). Thus, the L-PGDS/DP system is crucial for the homeostatic regulation of sleep.

An intraperitoneal administration of an L-PGDS inhibitor SeCl_4_ decreases the PGD_2_ concentration in the brain without changing the PGE_2_ and PGF_2α_ concentrations and induces complete insomnia during 1hr after the administration. *L-Pgds*, *L-Pgds*/*Hpgds* double or *Ptgdr* gene KO mice do not show the SeCl_4_-induced insomnia, whereas *Hpgds* or *Ptgdr2* gene KO mice show the SeCl_4_-induced insomnia, indicating that the insomnia depends on PGD_2_ produced by L-PGDS and recognizes by DP receptors (Qu et al., 2006). Pentylenetetrazole induces seizure in wild-type mice with a remarkable increase in the PGD_2_ concentration in the brain and induces excess sleep after seizure. The postictal sleep is not induced in *L-Pgds*, *L-Pgds*/*Hpgds* double or *Ptgdr* KO mice but observed in *Hpgds* and *Ptgdr2* KO mice, indicating that the sleep depends on the L-PGDS/PGD_2_/DP receptors system (Kaushik et al., 2014).

The SeCl_4_-induced insomnia is disappeared in leptomeninges-selective *L-Pgds* gene KO mice, which is generated by conditional gene depletion after injection of adeno-associated virus (AAV)-Cre vectors into the subarachnoidal space of newborn *L-Pgds* gene flox mice, but found in oligodendrocytes- or choroid plexus-specific conditional KO mice (Cherasse et al., 2018). These results indicate that the leptomeningeal L-PGDS is important for maintenance of physiological sleep.

Estradiol differentially regulates *L-Pgds* gene expression in the female mouse brain. Estradiol increases *L-Pgds* gene expression in the arcuate and ventromedial nucleus of the medial basal hypothalamus, a center of neuroendocrine secretions, and reduced in the ventrolateral preoptic area, a sleep center (Mong et al., 2003). Estradiol benzoate reduces *L-Pgds* gene expression in the sleep center and induces high motor activity in ovarectomized female mice (Ribeiro et al., 2009).

#### Protection of Neurons and Glial Cells

Lipocalin-type prostaglandin D_2_ synthase plays important roles for protection of neurons and glial cells under various pathological conditions. *L-Pgds* gene expression is upregulated in oligodendrocytes in *twitcher* mice as a model of human globoid cell leukodystrophy (Krabbe disease) caused by the mutation of galactosylceramidase (GALC). In double mutant of GALC^*twi/twi*^ *L-Pgds* gene KO mice, many neurons and oligodendrocytes exhibit apoptosis, indicating that L-PGDS protect neurons and glial cells against apoptotic loss cause by accumulation of cytotoxic glycosylsphingoid psychosine produced by the lack of GALC (Taniike et al., 2002). *L-Pgds* gene expression is upregulated in oligodendrocytes in mouse models of a variety of lysosomal storage disorders, such as Tay–Sachs disease, Sandhoff disease, GM_1_ gangliosidosis, and Niemann–Pick type C1 disease (Mohri et al., 2006a), suggesting that L-PGDS is involved in pathology of these diseases or may protect neurons and glial cells also in these diseases.

In a model of the hypoxic-ischemic encephalopathy of neonates, L-PGDS is induced as an early stress protein and protects neurons in neonatal mice (Taniguchi et al., 2007). *L-Pgds* gene KO mice exhibit greater infarct volume and brain edema after cerebral ischemia than wild type mice (Saleem et al., 2009). In neonatal rats, dexamethasone upregulates *L-Pgds* gene expression in the brain and protects hypoxic-ischemic brain injury. L-PGDS inhibitor SeCl4 or DP antagonist MK-0524 suppress the neuroprotective effect (Gonzalez-Rodriguez et al., 2014). In chronic intermittent hypoxia of adult rats, *L-Pgds* gene expression is increased in the brain from the second week (Shan et al., 2017). *L-Pgds* gene expression is upregulated in COX-2-overexpressing APP/PS1 mice, which exhibit more severe amyloid fibril formation than APP/PS-1 mice (Guan et al., 2019). These results suggest that L-PGDS is involved in the neuroprotection against the stress condition or the recovery from brain damage.

#### Amyloid β Clearance

Lipocalin-type prostaglandin D_2_ synthase binds Aβ (1–40) and Aβ (1–42) peptides at high affinities (Supplementary Table 4) and prevents their fibril formation *in vitro* (Kanekiyo et al., 2007; Kannaian et al., 2019). L-PGDS is a major CSF component responsible for prevention of Aβ fibril formation in human CSF *in vitro* (Kanekiyo et al., 2007). Infusion of Aβ (1–42) peptide into the brain attenuates and worsens, respectively, the Aβ fibril precipitation in the brain in *L-Pgds* gene KO and human *L-Pgds* gene-overexpressing TG mice, as compared with each wild-type mice (Kanekiyo et al., 2007). These results indicate that L-PGDS prevents Aβ fibril formation in the brain *in vivo*.

#### Depression-Related Behavior

Chronic stress via corticosterone treatment increases mRNA levels of COX-2 and L-PGDS in the brain. *Ptgdr2* gene-KO mice show antidepressant-like activity in a chronic corticosterone treatment-induced depression. The pharmacological inhibition of CRTH2 receptors in wild-type mice with a dual antagonist for CRTH2/TXA receptors, ramatroban, rescues depression-related behavior in chronic corticosterone-, LPS-, and tumor-induced pathologically relevant depression models. These results indicate that the L-PGDS/PGD_2_/CRTH2 axis is involved in progression of chronic stress-induced depression (Onaka et al., 2015).

#### Light-Induced Phase Advance

*L-Pgds* gene KO mice exhibit impaired light-induced phase advance, while they show normal phase delay and nonvisual light responses. *Ptgdr2* gene KO mice or CRTH2 antagonist CAY10471-administered wild-type mice also show impaired light-induced phase advance. These results indicate that L-PGDS is involved in a mechanism of light-induced phase advance via CRTH2 signaling (Kawaguchi et al., 2020).

#### Food-Intake

Several reports demonstrate the involvement of L-PGDS in food intake. Fasting upregulates *Ptgs2* and *L-Pgds* gene expression in the hypothalamus of mice, in which the orexigenic center exists. Intracerebroventricular administration of PGD_2_ or DP agonist stimulates food intake. DP antagonist, antisense oligonucleotide of DP receptors or an antagonist of neuropeptide Y (NPY) receptors suppress the orexigenic effects. Thus, the L-PGDS/DP/NPY axis regulates the food intake (Ohinata et al., 2008). Oral administration of a δ opioid peptide rubiscolin-6 stimulates food intake in wild-type and *Hpgds* gene KO mice, but not *L-Pgds* or *Ptgdr* gene KO mice. The orexigenic activity is found in *L-Pgds*^*flox*^/Nes-Cre mice, which lack *L-Pgds* gene in neurons and glial cells within the brain parenchyma but maintain *L-Pgds* gene in leptomeninges, choroid plexus and cerebroventricular ependymal cells. Thus, PGD_2_ produced by L-PGDS in leptomeningeal cells or cerebroventricular ependymal cells mediates the orexigenic effect (Kaneko et al., 2012). The activation of central δ-opioid receptor stimulates normal diet intake mediated by the orexigenic L-PGDS/PGD_2_/DP system but conversely suppresses high-fat diet intake through α-MSH/CRF pathway in a PGD_2_-independent manner (Kaneko et al., 2014).

### Lipocalin-Type Prostaglandin D_2_ Synthase in Peripheral Nervous System

Lipocalin-type prostaglandin D_2_ synthase and H-PGDS exist in neurons and glial cells, respectively, of chicken DRG (Vesin et al., 1995) and play an important role for maintenance of peripheral nervous system (PNS). *L-Pgds* gene KO mice exhibit hypomyelination of Schwann cells in PNS (Trimarco et al., 2014). In the primary culture, DRG neurons produce L-PGDS and PGD_2_, secrete these substances into the extracellular space, and stimulates myelination of Schwann cells. The L-PGDS inhibitor AT56 or gene knockdown of CRTH2 by shRNA suppresses the myelination of Schwann cells. Therefore, the L-PGDS/PGD_2_/CRTH2 axis plays important roles as a paracrine signal for development and maintenance of myelination of PNS (Trimarco et al., 2014). L-PGDS is necessary for macrophage activity and myelin debris clearance in a non-cell autonomous way during the resolution of PNS injury. In late phases of Wallerian degeneration, L-PGDS regulates the blood–nerve barrier permeability and SOX2 expression in Schwann cells, prevents macrophage accumulation, and exerts an anti-inflammatory role (Forese et al., 2020). Thus, L-PGDS has a different role during development and after injury in the PNS.

### Lipocalin-Type Prostaglandin D_2_ Synthase in Lung Inflammation

Prostaglandin D_2_ is an important inflammatory mediator involved in allergic asthma and L-PGDS is involved in the pro- and anti-inflammatory functions. Eosinophilic lung inflammation and Th2 cytokine release in ovalbumin-induced asthma model are enhanced in human *L-Pgds* gene-overexpressing TG mice (Fujitani et al., 2010). LPS or *Pseudomonas* treatment upregulates *L-Pgds* gene expression in the lung and alveolar macrophages. Removal of *Pseudomonas* from the lung is accelerated in the TG mice, or by intratracheal instillation of PGD_2_ to wild type mice, but impaired in *L-Pgds* gene KO mice. Thus, L-PGDS plays a protective role against the bacterial infection (Joo et al., 2007). L-PGDS inhibitor AT56 suppresses accumulation of eosinophils and monocytes in the broncho-alveolar lavage fluid in an antigen-induced asthma model of *Hpgds* gene KO mice. PGD_2_-produced by L-PGDS is involved in inflammatory cell accumulation in the alveolar lavage fluid (Irikura et al., 2009). Intratracheal administration of HCl results in lung inflammation accompanies by tissue edema and neutrophil accumulation. The deficiency of both *L-Pgds* and *Hpgds* genes exacerbates HCl-induced lung dysfunction to a similar extent. In this model, inflamed endothelial/epithelial cells express L-PGDS, while macrophages and neutrophils express H-PGDS. Vascular hyperpermeability in the inflamed lung is accelerated in *L-Pgds* gene KO mice and is suppressed by DP agonist. Thus, PGD_2_ is produced locally by L-PGDS in inflamed endothelial and epithelial cells and enhances the endothelial barrier through DP receptors (Horikami et al., 2019).

### Lipocalin-Type Prostaglandin D_2_ Synthase in Cardiovascular Function

*L-Pgds* gene is the most extensively expressed in the human heart (Eguchi et al., 1997) and L-PGDS plays cardioprotective function in several models. Estrogen induces *L-Pgds* gene expression in the heart of female mice by stimulating estrogen receptor (ER) β. An estrogen-responsive element in the *L-Pgds* gene promoter region is activated by ERβ, but not by ERα (Otsuki et al., 2003). Chronic hypoxia (10% O2) upregulates *L-Pgds* gene expression in the mouse heart. L-PGDS increases in the myocardium of auricles and ventricles and the pulmonary venous myocardium at 28 days of hypoxia. *L-Pgds* gene expression in the heart is two-fold more higher in heme oxygenase-2 KO mice, a model of chronic hypoxemia, than that of wild-type mice. Hypoxemia increases *L-Pgds* gene expression in the myocardium to adapt to the hemodynamic stress (Han et al., 2009).

Glucocorticoid stimulation with corticosterone or cortivazol induces calcium-dependent cPLA_2_, Cox-2 and L-PGDS in neonatal mouse cardiomyocytes. Glucocorticoids upregulate the expression of cPLA_2_, *Ptgs2* and *L-Pgds* genes and stimulate PGD_2_ synthesis in adult mouse heart. In isolated Langendorff-perfused mouse hearts, dexamethasone protects ischemia/reperfusion injury. Cox-2 inhibitor or depletion of *L-Pgds* gene completely suppresses the cardioprotective effect of dexamethasone. Dexamethasone reduces the infarct size by *in vivo* ischemia/reperfusion experiments of wild-type mice. The cardioprotective effect is markedly reduced in *L-Pgds* gene KO mice (Tokudome et al., 2009). Dexamethasone upregulates the expression of target genes characteristic erythroid-derived 2–like 2 (Nrf2) in wild-type but not *L-Pgds* gene-KO mice. Dexamethasone increases Nrf2 expression in an L-PGDS-dependent manner. Nrf2 KO mice do not show L-PGDS-mediated, dexamethasone-induced cardioprotection. Dexamethasone induces FP receptors in the mouse, rat and human heart. *Ptgfr* gene KO mice do not show the dexamethasone-induced cardioprotection. FP receptors bind PGD_2_ and PGF_2α_ with almost identical affinities. These results indicate that the dexamethasone-induced cardioprotective effect is mediated by the L-PGDS/PGD_2_/FP receptors axis through the Nrf2 pathway (Katsumata et al., 2014).

In perfused beating rat atria, hypoxia increases hypoxia-inducible factor (HIF) 1α, stimulates atrial natriuretic peptide (ANP) secretion, and upregulates *Ptgs2* and *L-Pgds* gene expression. HIF-1α antagonist, 2-methoxyestradiol, downregulates HIF-1α, *Ptgs2* and *L-Pgds* gene expression, and decreases hypoxia-induced ANP secretion. L-PGDS inhibitor AT-56 downregulates L-PGDS protein levels. The hypoxia-induced ANP secretion increases PPARγ protein levels and PPARγ antagonist GW9662 attenuates it. 2-Methoxyestradiol and AT-56 inhibit hypoxia-induced increase in atrial PPARγ protein. Thus, hypoxia activates the HIF-1α-L-PGDS-PPARγ signaling to promote ANP secretion in beating rat atria (Li et al., 2018). In the same model, acute hypoxia stimulates endothelin (ET)-1 release and expression of ET_*A*_ and ET_*B*_ receptors. ET-1 upregulates *Ptgs2* and *L-Pgds* gene expression and increases PGD_2_ production through activation of ET_*A*_ and ET_*B*_ receptors. L-PGDS-derived PGD_2_ promotes hypoxia-induced ANP secretion and in turn regulates *L-Pgds* gene expression by an Nrf2-mediated feedback mechanism. ET-1 induced by hypoxia activates the Cox-2/L-PGDS/PGD_2_ signaling and promotes ANP secretion. The positive feedback loop between L-PGDS-derived PGD_2_ and hypoxia-induced *L-Pgds* gene expression is a part of the mechanism of hypoxia-induced ANP secretion by ET-1 (Li et al., 2019).

### Lipocalin-Type Prostaglandin D_2_ Synthase in Obesity and Adipocyte Differentiation

A high-fat diet (HFD) upregulates *L-Pgds* gene expression in adipose tissues and differentiates adipocytes (Fujimori et al., 2007a, 2012b; Chowdhury et al., 2011) so that *L-Pgds* gene KO mice show several abnormalities in the regulation of energy homeostasis. *L-Pgds* gene KO mice exhibit glucose-intolerant and insulin-resistant at an accelerated rate as compared with wild-type mice. Adipocytes are larger in *L-Pgds* gene KO mice than those of control mice with the same diet. *L-Pgds* gene KO mice develop nephropathy and an aortic thickening reminiscent to the early stages of atherosclerosis when fed HFD (Ragolia et al., 2005). Adipocytes of *L-Pgds* gene KO mice are less sensitive to insulin-stimulated glucose transport than those of wild-type mice (Ragolia et al., 2008).

*L-Pgds* gene KO mice exhibit body weight gain more than WT mice when fed HFD, and increase subcutaneous and visceral fat tissues. HFD-fed *L-Pgds* gene KO mice exhibit increased fat deposition in the aortic wall, atherosclerotic plaque in the aortic root, macrophage cellularity and the expression of pro-inflammatory cytokines such as IL-1β and monocyte chemoattractant protein-1. Thus, *L-Pgds* gene deficiency induces obesity and facilitates atherosclerosis through the regulation of inflammatory responses (Tanaka et al., 2008).

*L-Pgds* gene KO mice display features of the metabolic syndrome in the absence of HFD as well as with HFD feeding and this correlates with hyperactivity of the hypothalamus-pituitary-adrenal (HPA) axis, i.e., increases in plasma ACTH and corticosterone concentrations, at 20-week-old. C57BL/6 mice exhibit age-related increases in HPA activity, whereas *L-Pgds* gene KO mice are resistant to changes in HPA activity with age and long-term HFD feeding. Thus, these events depend on *L-Pgds* gene expression (Evans et al., 2013).

Vertical sleeve gastrectomy (VSG) is used to improve metabolic complications in patients with obese and diabetes. VSG improves glycemic parameters 10 weeks after operation in WT and *L-Pgds* gene knock-in (KI) mice but not in *L-Pgds* gene KO mice, as compared with the sham-operated group. *L-Pgds* gene KO mice develop glucose intolerance and insulin resistance even after VSG similar to or greater than the sham group. *L-Pgds* gene KO mice exhibit post-VSG peptide YY levels slightly increased but significantly less than other groups and the leptin sensitivity in response to VSG less than KI mice. Total cholesterol level is unchanged in all groups irrespective of sham or VSG surgery. However, *L-Pgds* gene KO mice show higher cholesterol levels and increase the adipocyte size in post-VSG. Thus, L-PGDS plays an important role in the beneficial metabolic effects by VSG (Kumar et al., 2016).

*L-Pgds* gene KO mice show hypertension and acceleration of thrombogenesis but *Hpgds* gene KO mice do not change these functions (Song et al., 2018). Therefore, L-PGDS is also important for the control of blood pressure and thrombosis.

There are two distinct types of adipose-specific *L-Pgds* gene KO mice: one is fatty acid binding protein 4 (fabp4, aP2)-Cre/*L-Pgds*^ flox/flox^ mice and the other is adiponectin (AdipoQ)-Cre/*L-Pgds*^ flox/flox^ mice. The former strain lacks the *L-Pgds* gene in adipocytes even in the premature stage and the latter strain, only after maturation. The former strain decreases *L-Pgds* gene expression and PGD_2_ production levels in white adipose tissue under HFD conditions, whereas the latter strain does not change the L-PGDS and PGD_2_ levels. When fed an HFD, the former strain aP2-Cre/*L-Pgds*^ flox/flox^ mice reduce body weight gain, adipocyte size, and serum cholesterol and triglyceride levels. In white adipose tissue of HFD-fed aP2-Cre/*L-Pgds*^ flox/flox^ mice, the expression levels of adipogenic, lipogenic, and M1 macrophage marker genes are decreased, whereas the lipolytic and M2 macrophage marker genes are enhanced or unchanged. Insulin sensitivity is improved in HFD-fed aP2-Cre/*L-Pgds*^ flox/flox^ mice. Therefore, PGD_2_ produced by L-PGDS in premature adipocytes is involved in the regulation of body weight gain and insulin resistance under nutrient-dense conditions (Fujimori et al., 2019).

In PPARγ-KO mice, *L-Pgds* gene expression is increased in brown adipose tissue (BAT) and subcutaneous white adipose tissue but reduced in the liver and epididymal fat (Virtue et al., 2012b). Double KO mice of PPARγ and *L-Pgds* genes exhibit reduced expression of thermogenic genes, the *de novo* lipogenic program and the lipases in subcutaneous white adipose tissue but elevated expression of lipolysis genes in epididymal fat. These results indicate that PPARγ and L-PGDS coordinate to regulate carbohydrate and lipid metabolism (Virtue et al., 2012b). In BAT, *L-Pgds* gene expression increases after HFD feeding or cold exposure at 4C (Virtue et al., 2012a). The L-PGDS induction depends on PGC1α and 1β, positive regulators of BAT activation, and is reduced by RIP140, a negative regulator of BAT activation. Under cold–acclimated conditions, *L-Pgds* gene KO mice exhibit elevated reliance on carbohydrate used for thermogenesis and increase expression of genes regulating glycolysis and *de novo* lipogenesis in BAT.

In *ob/ob* mice, *L-Pgds* gene expression is decreased in white adipose tissue whereas *Hpgds* gene expression is markedly increased. In white adipose tissue, H-PGDS exists in macrophages and is involved in polarization of macrophages toward to M2, anti-inflammatory, state in both mice and human (Virtue et al., 2015).

### Lipocalin-Type Prostaglandin D_2_ Synthase in Bone and Cartilage Metabolism

In the mouse model of collagen-induced arthritis (CIA), PGD_2_ is produced in the joint during the early phase. Serum PGD_2_ levels increase progressively throughout the arthritic process and reach to a maximum during the late stages. The expression of *L-Pgds*, *Hpgds*, *Ptgdr*, and *Ptgdr2* genes increases in the articular tissues during arthritic process. DP antagonist MK0524 increases the incidence and severity of CIA, and the local levels of IL-1β, CXCL-1, and PGE_2_ but reduces the IL-10 levels. CRTH2 antagonist CAY10595 does not modify the severity of arthritis. The administration of PGD_2_ or a DP agonist BW245C reduces the incidence of CIA, the inflammatory response, and joint damage. In the articular tissue during development of CIA, PGD_2_ plays an anti-inflammatory role through DP receptors (Maicas et al., 2012). In the spontaneous Hartley guinea pig and experimental dog arthritis models, *L-Pgds* gene expression also increases over the course of osteoarthritis. In the guinea pig model, L-PGDS levels are correlated positively with the histological score of osteoarthritis (Nebbaki et al., 2013).

In an experimental osteoarthritis model induced by destabilization of the medial meniscus, *L-Pgds* gene KO mice exhibit exacerbated cartilage degradation and enhanced expression of matrix metalloproteinase 13 (MMP-13) and a disintegrin and metalloprotein-ase with thrombospondin motifs 5 (ADAMTS-5), and display increased synovitis and subchondral bone changes (Najar et al., 2020). Cartilage explants from *L-Pgds* gene KO mice show enhanced proteoglycan degradation after treatment with IL-1α. Intra-articular injection of AAV2/5 encoding *L-Pgds* gene attenuates the severity of osteoarthritis in wild-type mice, by an increase in L-PGDS level in the osteoarthritis tissue. *L-Pgds* gene KO mice show the accelerated development of naturally occurring age-related osteoarthritis (Ouhaddi et al., 2020). *L-Pgds* gene deletion promotes cartilage degradation during aging and enhances expression of extracellular matrix degrading enzymes, MMP-13 and ADAMTS-5, and their breakdown products. Moreover, *L-Pgds* gene deletion enhances subchondral bone changes without effect on its angiogenesis, increases mechanical sensitivity, and reduces spontaneous locomotor activity. *L-Pgds* gene expression increases in aged mice, suggesting that L-PGDS plays an important role to protect against naturally occurring age-related osteoarthritis. L-PGDS may be a new efficient therapeutic target in osteoarthritis.

### Lipocalin-Type Prostaglandin D_2_ Synthase in Keratinocytes and Hair Follicle Neogenesis

In a wound-induced hair follicle neogenesis model, L-PGDS and PGD_2_ levels of the skin are negatively correlated with the hair follicle neogenesis among C57Bl/6J, FVB/N, and mixed strain mice (Nelson et al., 2013). The hair follicle regeneration increases in mice with an alternatively spliced transcript variant of *L-Pgds* gene without exon 3 and *Ptgdr2* gene KO mice, but not in *Ptgdr* gene KO mice. Keratinocytes produce L-PGDS in the skin. PGD_2_ produced by L-PGDS in keratinocytes inhibits wound-induced hair follicle neogenesis through CRTH2 receptors.

### Lipocalin-Type Prostaglandin D_2_ Synthase in Mast Cell Differentiation and Anaphylaxis

Mast cells play important roles in anaphylaxis. Phenotypes of mast cells are changed by microenvironment. Mast cells dominantly express H-PGDS (Urade et al., 1990) and release PGD_2_ after anti-IgE stimulation. For maturation of bone-marrow derived mast cells *in vitro*, it is necessary for immature mast cells to interact with fibroblasts, suggesting that fibroblasts release some mediators for maturation of mast cells. The mediator is PGD_2_ produced by L-PGDS in fibroblasts1. Mast cells secrete phospholipase A_2_ (PLA2G3), which couples to the Cox/L-PGDS system in fibroblasts to produce PGD_2_. PGD_2_ released from fibroblasts then stimulates DP receptors on immature mast cells to promote their maturation (Taketomi et al., 2013).

### Lipocalin-Type Prostaglandin D_2_ Synthase in Colon

In experimental colitis model with dextran sodium sulfate in the drinking water, the disease activity reduces in *L-Pgds* gene KO mice than WT mice (Hokari et al., 2011). PGD_2_ derived from L-PGDS plays pro-inflammatory roles in the dextran-induced colitis.

### Lipocalin-Type Prostaglandin D_2_ Synthase in Adenoma

In tumor generation in *Apc*^*Min/*+^ mice mated with various mutations of *L-Pgds*, *Hpgds* and *Ptgdr* genes, adenoma production is enhanced in KO mice of *Hpgds* or *Ptgdr* gene but slightly reduced in *L-Pgds* gene KO mice. *Apc*^*Min/*+^ mice overexpressing human *Hpgds* or *L-Pgds* gene suppress the tumor generation (Tippin et al., 2014).

### Lipocalin-Type Prostaglandin D_2_ Synthase in Melanoma

Lipocalin-type prostaglandin D_2_ synthase is expressed in endothelial cells of human melanoma and oral squamous cell carcinoma (Omori et al., 2018). Human endothelial cells produce L-PGDS and PGD_2_ after stimulation with IL-1 and TNFα derived from tumor cells. Melanoma growth is accelerated in *L-Pgds* gene KO mice or endothelial cell-specific *L-Pgds* gene KO mice and is attenuated by administration of a DP agonist BW245C. *L-Pgds* gene deficiency in endothelial cells accelerates vascular hyperpermeability, angiogenesis, and endothelial-mesenchymal transition in tumors to reduce tumor cell apoptosis. Tumor cell-derived inflammatory cytokines increase *L-Pgds* gene expression and PGD_2_ production in tumor endothelial cells. PGD_2_ is a negative regulator of the tumorigenic changes in tumor endothelial cells.

### Lipocalin-Type Prostaglandin D_2_ Synthase in Renal Function

In a mouse model of Adriamycin-induced nephropathy, L-PGDS is induced in tubules including proximal, Henle’s loop and distal compartments of the kidney (Tsuchida et al., 2004). Urinary L-PGDS excretion increases from day 1 onward, and apparently precedes the increase in urinary albumin excretions. Neither serum L-PGDS nor creatinine levels are changed by administration of Adriamycin. However, serum creatinine levels are inversely correlated to urinary L-PGDS excretions. The urinary L-PGDS is a useful marker of renal permeability dysfunction.

Otsuka Long-Evans Tokushima Fatty (OLETF) rats develop diabetes associated with hypertension and exhibit higher urinary L-PGDS excretion than non-diabetic Long-Evans Tokushima Otsuka rats (Ogawa et al., 2006). The urinary L-PGDS excretion in OLETF rats increases in an age-dependent manner and is due to increased glomerular permeability to L-PGDS. Renal tissue contains L-PGDS mRNA and immunoreactivity. However, glomerular filtration of L-PGDS contributes to urinary L-PGDS excretion much more than the *de novo* synthesis. Multiple regression analysis shows that urinary L-PGDS is determined by urinary protein excretions and not by high blood pressure *per se.* Conversely, the urinary L-PGDS excretion in the early stage of diabetes predicts the urinary proteinuria in the established diabetic nephropathy.

Crude extracts of monkey kidney and human urine contain L-PGDS isoforms with its original N-terminal sequence starting from Ala23 after the signal sequence, and from Gln31 and Phe34 with its N-terminal-truncation (Nagata et al., 2009). The mRNA and the intact form of L-PGDS exist in the cells of Henle’s loop and the glomeruli of the kidney, as examined by *in situ* hybridization and immunostaining with monoclonal antibody 5C11, which recognizes the amino-terminal loop from Ala23 to Val28 of L-PGDS. Those cells and tissues produce L-PGDS *de novo* within the kidney. Truncated forms of L-PGDS exist in the lysosomes of tubular cells, as visualized by immunostaining with monoclonal antibody 10A5, which recognizes the 3-turn α-helix between Arg156 and Thr173 of L-PGDS. Therefore, tubular cells uptake L-PGDS and degrade within lysosomes to produce the truncated form.

Lipocalin-type prostaglandin D_2_ synthase contributes to the progression of renal fibrosis via CRTH2-mediated activation of Th2 lymphocytes (Ito et al., 2012). In a mouse model of renal fibrosis caused by unilateral ureteral obstruction, the tubular epithelium produces L-PGDS *de novo*. *L-Pgds* or *Ptgdr2* gene KO mice exhibit less renal fibrosis, reduce infiltration of Th2 lymphocytes to the cortex, and decrease production of Th2 cytokines IL-4 and IL-13. Administration of a CRTH2 antagonist Cay10471 at 3 days after the obstruction suppresses the progression of renal fibrosis. IL-4- or IL-13 KO mice also ameliorate the kidney fibrosis in the unilateral obstruction model. Blocking of CRTH2 receptors may be useful to slowdown the progression of renal fibrosis in chronic kidney diseases.

### Lipocalin-Type Prostaglandin D_2_ Synthase in Preterm Birth

LPS-induced preterm birth occurs 89% and 100%, respectively, in C57BL/6 mice and *L-Pgds* gene-overexpressing TG mice and significantly reduced to 40% in *L-Pgds* gene KO mice (Kumar et al., 2015). Administration of DP or CRTH2 antagonists to C57BL/6 mice increases the number of viable pups 3.3-fold, indicating that PGD_2_-mediated inflammation is involved in the preterm birth and the dead birth.

### Lipocalin-Type Prostaglandin D_2_ Synthase in Uterus

Double KO mice of both *L-Pgds* and *Hpgds* genes develop adenomyotic lesions in the uterus at 6-month-old (Philibert et al., 2021). The disease severity increases with age, suggesting that the PGD_2_ signaling has major roles in the uterus by protecting the endometrium against development of adenomyosis.

### Lipocalin-Type Prostaglandin D_2_ Synthase in Testis and Epididymis

Male genital organs highly express L-PGDS in various animals including humans (Tokugawa et al., 1998; Gerena et al., 2000). The regional distribution and regulation of L-PGDS expression are examined in rat testis and epididymis by *in situ* hybridization and immunohistochemistry under the conditions of sexual maturation, castration, and ethylene dimethane sulfonate treatments, which eliminates Leydig cells in the testicular interstitium (Zhu et al., 2004). In sexually mature rats, testicular peritubular cells weakly expressed L-PGDS but Leydig cells in the testis highly expressed L-PGDS by day 70 postpartum. L-PGDS is highly detected in the caput, corpus, and cauda of the epididymis during sexual maturation 70 days after birth. Castration and ethylene dimethane sulfonate treatments significantly decrease L-PGDS expression in the epididymis. Testosterone propionate treatment increases L-PGDS expression in the epididymis of both castrated and ethylene dimethane sulfonate-treated rats. Testosterone up-regulates *L-Pgds* gene expression in rat epididymis.

Both heterozygous and homozygous *L-Pgds* gene KO mice present unilateral cryptorchidism affecting the second phase of testicular descent in 16% and 24% of cases, respectively (Philibert et al., 2013). The adult cryptorchid testes increases the spermatogonia apoptosis and decreases the global tubule size parameters. The gubernaculum of newborn mutants shows some histological abnormalities. In 29 children with cryptorchidism, none of the investigated cases presented mutations in the *L-Pgds* gene. L-PGDS is a novel component in the cryptorchidism (Philibert et al., 2013).

### Lipocalin-Type Prostaglandin D_2_ Synthase in Prostate Gland

Administration of a low dose of bisphenol A to male SD rats induces prostatic hyperplasia and upregulates *Ptgs2* and *L-Pgds* gene expression in the prostate. The prostatic hyperplasia is suppressed by administration of inhibitors for Cox-2 and L-PGDS with increased apoptosis levels, indicating that Cox-2 and L-PGDS mediate low-dose bisphenol A-induced prostatic hyperplasia (Wu et al., 2020).

### Lipocalin-Type Prostaglandin D_2_ Synthase in Male Germ Cell Differentiation in the Fetal Testis

The male/female ratio and the number of infant are unchanged among WT, KO mice of *L-Pgds*, *Hpgds*, *ptgdr* and *ptgdr2* genes, and double KO mice of *L-Pgds* and *Hpgds* genes. However, the delayed development of fetal mouse testis occurs in several KO lines, indicating that PGD_2_ produced by L-PGDS and H-PGDS is involved in differentiation of somatic and germ cells in testis by stimulating DP and CRTH2 receptors, respectively. The role of the PGD_2_ signaling pathway in reproduction was reviewed (Rossitto et al., 2015). L-PGDS is one of the most male-enriched gene transcripts expressed at 12.5 days post coitum (dpc) in an early stage of mouse embryonic gonads (Adams and McLaren, 2002). The time course and place of L-PGDS expression in mouse embryo are similar to those of the sex-determining factor Sox9. In embryonic gonads, L-PGDS exists only in male XY gonads but not in female XX gonads. L-PGDS is expressed downstream of Sox9. PGD_2_ is an autocrine factor inducing Sox9 nuclear translocation after DP receptor-mediated cAMP-PKA phosphorylation, which induces subsequent Sertoli cell differentiation (Malki et al., 2005). Sox9 dimer binds to a paired Sox/Sry binding site in a promoter region of the L-PGDS gene and trans-activates the male-specific L-PGDS gene expression (Wilhelm et al., 2007) to make a positive feed-back loop of the L-PGDS-Sox9 signaling. In Sox9 KO mice, L-PGDS disappears in embryonic Sertoli cells of the XY gonads. In L-PGDS KO mice, the Sox9 transcript decreases in Sertoli cells of the XY embryonic gonad and the subcellular localization of Sox9 protein is perturbed (Moniot et al., 2009). H-PGDS exists in somatic and germ cells of the embryonic gonad of both sexes at embryonic day 10.5, before the onset of L-PGDS expression. Administration of H-PGDS inhibitor HQL79 to WT mice suppresses the nuclear translocation of Sox9 protein in embryonic Sertoli cells. In *Hpgds* gene KO mice, Sox 9 protein remains mainly within the cytoplasm of embryonic Sertoli cells at the early stage of mouse testicular differentiation. H-PGDS-produced PGD_2_ is involved in the initial nuclear translocation of Sox9 during the early stage of testicular differentiation (Moniot et al., 2011). In double KO mice of *L-Pgds* and *Hpgds* genes, i.e., depleted for PGD_2_, and *ptgdr2* gene KO mice, a significant proportion of the germ cells are not mitotically arrested and still engaged in the cell cycle at a time which should be quiescent (Moniot et al., 2014). Germ cells of those KO mice downregulate cell cycle inhibitors p21^*Clip1*^ and p57^*Kip2*^ and upregulate cell cycle activators CyclinE1 and E2. Therefore, the PGD_2_ signaling is involved in the control of cell cycle genes in fetal testis to arrest mitotic process. At late embryonic stages of those KO mice, the ectopic expression of pluripotency markers Pou5f1 (Oct4), Sox2 and Nanog is detected in the testis and the male germ cell marker Nanos2 is downregulated. The PGD_2_ system is also involved in germ cell differentiation in the embryonic testis. Somatic factor Cyp26B1 is a retinoic acid–metabolizing enzyme of P450 family produced by Sertoli cells. Cyp26B1 protects germ cells from retinoic acid. The mutant gonads reduce Cyp26B1. Thus, PGD_2_ produced by Sertoli cells influences the differentiation of the embryonic germ cells.

The L/H-PGDS/PGD_2_ system is important for sex organ development. Therefore, the *in utero* exposure to NSAIDs, such as acetaminophen and ibuprofen, during the sex determination period results in malfunction of both male and female genital organs and also intergenerational subfertility (Rossitto et al., 2019a, b).

## Pharmacokinetic Analyses and Functionalization with Recombinant Lipocalin-Type Prostaglandin D_2_ Synthase

Pharmacokinetic analyses with recombinant L-PGDS after an intravenous injection to dogs (Li et al., 2008) reveal that the serum concentration of L-PGDS decreases with t1/2 of 0.77 h, which is shorter than that of other proteins with the same molecular mass as that of L-PGDS. Only about 10% of administered L-PGDS is recovered into the urine. Thus, majority of L-PGDS seems to be taken up by the tissues and/or degraded within the body. After the intrathecal injection, about one third of the administered L-PGDS is excreted to the blood, suggesting that L-PGDS is degraded or taken up by neural cells within the CNS or excreted through the lymphatic fluid.

Lipocalin-type prostaglandin D_2_ synthase is useful for functionalization of nanoparticles to increase the brain-blood barrier permeability and intracellular uptake efficiencies (Portioli et al., 2017). L-PGDS-conjugated nanoparticles are taken up by neurons and glial cells mediated by an LDL receptor-mediated mechanism after administration from the tail vein of mice. This report also suggest that other cells uptake L-PGDS through LDL receptors as an intercellular transporter of various lipophilic substances. The intercellular transport of L-PGDS may be involved in the finding that L-PGDS protein injected into the mouse brain promotes migration and accumulation of astrocytes *in vivo* (Lee et al., 2012).

## Studies of Nonmammalian Orthologs of Lipocalin-Type Prostaglandin D_2_ Synthase

There are many studies of L-PGDS in non-mammals, including birds, amphibians and fishes.

In chicken DRG, the L-PGDS-immunoreactivity is detected in neurons and the H-PGDS-immunoreactivity, in glial cells (Vesin et al., 1995). cDNA for the chicken ortholog of L-PGDS is identified (Fujimori et al., 2006). The recombinant chicken L-PGDS is associated with the weak PGD_2_ synthase activity of about 3% of that of the mouse enzyme and possesses the binding activities of retinoic acid and thyroxine with almost comparable affinities (Kd = 0.6–0.7 μM) to those of the mouse enzyme (Fujimori et al., 2006). The chicken L-PGDS is also expressed in a male specific mechanism regulated by Sox9 (Moniot et al., 2008), similar to mouse L-PGDS.

The amphibian ortholog of L-PGDS is identified in three different species of frogs, *Xenopus laevis*, Cane Todd (*Bufo marinus*) and Japanese treefrog (*Hyla japonica*) (Irikura et al., 2007). The recombinant protein of toad L-PGDS is associated with the weak PGD_2_ synthase activity of about 4% of that of the rat enzyme and possesses the binding activities of bilirubin, biliverdin and retinoic acid with a weak affinity (Kd = 2 μM) and of thyroid hormones with almost comparable affinities (Kd = 0.9–1.6 μM) to those of the rat enzyme (Irikura et al., 2007). The toad L-PGDS exists in choroid plexus and is secreted into the CSF. *Xenopus* L-PGDS is one of the genes activated by Zic1, a transcription factor expressed at the anterior neural plate to promote the placode fate of embryo, and act as a retinoic acid-transporter directly participate in the establishment of the pre-placodal region (Jaurena et al., 2015).

The fish ortholog of L-PGDS is found in Zebrafish (Fujimori et al., 2006). The fish L-PGDS ortholog is not associated with the PGD_2_ synthase activity due to the loss of the active site Cys65 residue but maintains the binding affinities for thyroxine and *all-trans-*retinoic acid (Kd = 0.4–1.0 μM), like mammalian L-PGDSs. In Catfish, the L-PGDS ortholog is expressed in seminal vesicle, in which the *L-Pgds* gene expression is decreased by L-thyroxin overdose and increased by depletion of thyroid hormone, suggesting that the L-PGDS protein is involved in the thyroid hormone pathway even in fish (Sreenivasulu et al., 2013).

## Clinical Studies of Pathophysiological Function of Lipocalin-Type Prostaglandin D_2_ Synthase

In human tissues, L-PGDS mRNA expression is most intense in the heart following the brain, and widely and weakly detected in many other organs, such as the placenta, lung, liver, skeletal muscle, kidney, pancreas, and others (Eguchi et al., 1997). Clinical studies of patients with various types of diseases reveal a variety of pathophysiological function of L-PGDS, as summarized in Supplementary Table 7.

### Central Nervous System Function

#### Sleep Regulation

In the CNS, L-PGDS is involved in sleep regulation (Urade and Hayaishi, 2011). Sleep attack or coma caused by overproduction of PGD_2_ is reported in patients with mastocytosis (Roberts et al., 1980) and African sleeping sickness (Pentreath et al., 1990). Under normal physiological condition, serum L-PGDS levels increase in evening and decrease by total sleep deprivation but not by partial sleep deprivation selective to rapid eye movement sleep (Jordan et al., 2004). CSF L-PGDS levels in day time (10:00 AM – 1:00 PM) are lower in patients with excess daytime sleepiness (EDS) than control healthy volunteers (Bassetti et al., 2006). Narcolepsy is a sleep disorder associated with EDS in daytime and caused by degeneration of hypothalamic neurons which produce a neuropeptide hypocretin/orexin. CSF and serum levels of L-PGDS are higher in patients with narcolepsy than control patients (Jordan et al., 2005; Wang et al., 2020). CSF and serum levels of L-PGDS are also higher in patients with ideopathic hypersomnia without degeneration of hypocretin/orexin-producing neurons than control patients (Wang et al., 2020). In patients with obstructive sleep apnea (OSA), serum L-PGDS levels are higher in patients with EDS than those without EDS or controls (Barceló et al., 2007). Urinary L-PGDS levels are higher in patients with severe OSA than those in control or moderate OSA. The urinary excretion of severe OSA patients decreases to the control level after continuous positive airway pressure treatment to maintain the respiration during sleep (Chihara et al., 2013). The L-PGDS/β-trace concentrations in CSF, serum and urine are positively related with sleepiness in many sleep disorders.

#### Food Intake

Cerebrospinal fluid L-PGDS levels are positively related with food intake in a weight loss study with a 3-week dietary lead-in followed by 12-weeks of leptin or placebo treatment (Elias et al., 2011). CSF L-PGDS levels at the base line are related positively with NPY, galanin, visceral adipose tissue volume, corticotropin-releasing hormone and beta-endorphin, and inversely with CSF leptin. Leptin treatment does not affect CSF L-PGDS and NPY levels. Serum L-PGDS levels are not related with any of the measured variables either at baseline or after leptin treatment.

#### Cerebrospinal Fluid Circulation

Cerebrospinal fluid levels of L-PGDS are altered in malfunction of CSF circulation. Normal pressure hydrocephalus exhibits an abnormal CSF flow in the ventricles or cavities within the brain. This disease is caused by blocking CSF flow throughout the brain and spinal cord. Patients exhibit progressive mental impairment and dementia, problems with walking, and impaired bladder control. CSF L-PGDS levels decrease in patients with this disease (Mase et al., 2003) in disproportionately enlarged subarachnoid-space (Nishida et al., 2014) and show a trend of increase in the cognitive-improved patients after lumboperitoneal shunting but not in the poor cognitive-improved patients (Nakajima et al., 2015).

Spontaneous intracranial hypotension is a secondary headache etiology caused by CSF leakage, in which CSF L-PGDS levels are higher than control (Murakami et al., 2018).

Lipocalin-type prostaglandin D_2_ synthase is useful to monitor the CSF drainage from human brain (Tsutsumi et al., 2015). The diploic vein/peripheral vein ratio of L-PGDS concentrations increases in the frontal, temporal, parietal and skull base. The diploic vein/peripheral vein ratio decreases in the frontal region for patients older than 45 years. The diploic veins constitute CSF drainage pathways with heterogeneous function intensity at different cranial locations.

#### Neurodegenerative Diseases

Induction of L-PGDS in neurons and glial cells is found in several neurodegenerative diseases. CSF L-PGDS levels are unchanged in patients with multiple sclerosis (Kagitani-Shimono et al., 2006). However, as examined with autopsy samples from patients with multiple sclerosis, L-PGDS immunoreactivity increases in oligodendrocytes within the shadow plaques and in hypertrophied astrocytes within the chronic plaques (Kagitani-Shimono et al., 2006). In neonatal hypoxic-ischemic encephalopathy, the surviving neurons in the infarcted lesions express L-PGDS immunoreactivity (Taniguchi et al., 2007).

After subarachnoid hemorrhage, CSF L-PGDS levels increase ∼two-fold at days 3 and 5, and return to the basal level at day 17 (Mase et al., 1999). L-PGDS in CSF from those patients covalently binds biliverdin, a by-product of heme breakdown, at Cys65 residue. L-PGDS scavenges harmful heme-degradation products in CSF after subarachnoid hemorrhage (Inui et al., 2014). Therefore, L-PGSDS acts as an extracellular scavenger of lipophilic toxic substance in CSF.

### Cardiovascular Diseases

In human heart, L-PGDS exists in myocardiocytes, atrial endocardiocytes and a synthetic phenotype of smooth muscle cells in the arteriosclerotic intima, as examined by immunostaining of autopsy specimens (Eguchi et al., 1997). L-PGDS also exists in the atherosclerotic plaque of coronary arteries with severe stenosis. In patients with stable angina, the plasma level of L-PGDS is higher in the great cardiac vein than in the coronary artery. Therefore, the stenotic site produces and secrets L-PGDS to the coronary circulation (Eguchi et al., 1997). In patients undergoing percutaneous transluminal coronary angioplasty, L-PGDS levels in coronary sinus blood at 48 h after the treatment reduce to the baseline level in patients with restenosis but increase in those without restenosis (Inoue et al., 2001).

Serum L-PGDS levels are altered by many vascular diseases. Hypertensive patients show the increased serum L-PGDS levels with the renal function worsened. The urinary excretion is higher in hypertensive patients than normotensive patients (Hirawa et al., 2002). There is a common SNP of 4111 A>C in 3′-untranslated region of the L-PGDS gene in Japanese. Serum levels of HDL cholesterol are higher in hypertensive subjects with the A/A genotype than those with the A/C and C/C genotypes. The subjects with the A/A genotype exhibit the maximum intima-media thickness in the common carotid artery smaller than those with the A/C and C/C genotypes (Miwa et al., 2004). Serum L-PGDS levels increase in associated with the progression of atherosclerosis in non-treated asymptomatic subjects of atherosclerosis (Miwa et al., 2008). Serum L-PGDS levels are powerful biomarkers of severity of stable coronary artery disease in patients with coronary angiography (Inoue et al., 2008). Serum L-PGDS levels are higher in patients with vasospastic angina, and negatively correlated with the degree of the left anterior descending coronary artery vasomotion in response to acetylcholine (Matsumoto et al., 2011). Serum L-PGDS levels are positively correlated with cardiovascular diseases in Japanese.

On the other hand, in a study with gender- and age-matched Scandinavian individuals, serum L-PGDS is unchanged between patients with or without cardiovascular disease, while osteoprotegerin concentrations increase in patients with acute coronary syndrome (Hosbond et al., 2014). Pathological changes in serum L-PGDS levels may be different among populations with different genetics and nutrient circumstances.

Serum L-PGDS levels are higher in the pulmonary venous blood in patients with pulmonary embolism than control (Mutlu et al., 2020).

### Metabolic Syndrome

Serum L-PGDS levels are associated with hypertriglyceridemia but not diabetes in patients with metabolic syndrome (Cheung et al., 2013).

### Renal Function

Urinary L-PGDS levels are useful marker of renal function. Urinary L-PGDS excretion increases in the early stage of kidney injury in patients with type-2 diabetes mellitus (Hirawa et al., 2001) and is reversed by blood sugar control (Hamano et al., 2002). L-PGDS appears in the renal tubules in diabetes patients but does not exist in nondiabetic patients (Hamano et al., 2002). Urinary L-PGDS levels in type 2 diabetes patients are related with cardiovascular disease and useful as a supplemental or additional marker to the criteria of metabolic syndrome (Yoshikawa et al., 2007). A prospective study for ∼2 years with 121 patients of type-2 diabetes with <30 mg/g Cr albuminuria reveals that urinary L-PGDS levels are useful to predict the future status of renal injury in those patients (Uehara et al., 2009).

Urinary L-PGDS levels increase by the renal injury after long-term administration of gentamycin (Nakayama et al., 2009). In patients with systemic lupus erythematosus, urinary L-PGDS levels are significantly higher with active vs. inactive lupus nephritis or in patients without lupus nephritis. Urinary L-PGDS excretion increases as 3 months before a clinical diagnosis of worsening lupus nephritis (Suzuki et al., 2009) and decreases in patients with mucopolysaccharidosis type II disease (Hunter disease) as compared with age- and gender-matched healthy controls (Yuan et al., 2019).

### Cancers

Lipocalin-type prostaglandin D_2_ synthase expression is different among the types of cancers. L-PGDS exists in tumor cells of all various types of ovarian cancers (Su et al., 2001) but not in lung tumors (Ragolia et al., 2010). L-PGDS expression increases in malignant melanomas (Shimanuki et al., 2012), and is negatively correlated with Yes-associated protein 1 (YAP) in gastric cancers (Bie et al., 2020).

### Bone and Cartilage

Lipocalin-type prostaglandin D_2_ synthase is induced in cartilages of patients with osteoarthritis, suggesting that L-PGDS has an important role in the pathophysiology of OA (Zayed et al., 2008).

### Digestive Tract

Lipocalin-type prostaglandin D_2_ synthase is involved in inflammation of digestive tract. L-PGDS is induced in fibroblasts close to infiltrating cells in *Helicobacter pylori*-infected gastric mucosa (Hokari et al., 2009), and lamina proprial infiltrating cells and muscularis mucosa in patients with ulcerative colitis in parallel with the disease activity (Hokari et al., 2011). Patients with Crohn’s disease show L-PGDS and Cox-2 mRNA expressions and increased PGD_2_ levels in inflamed colonic mucosa at the active stage (Le Loupp et al., 2015). L-PGDS appears in neurons of both myenteric and submucosal plexi of the patients with Crohn’s disease (Le Loupp et al., 2015).

### Inflammation

In clinically healthy 58-year-old 100 Swedish men, serum L-PGDS levels positively correlate with soluble TNF-receptors 1 and 2 and negatively with alcohol consumption and serum HDL but not with insulin sensitivity (Wallenius et al., 2011). These results suggest that serum L-PGDS acts as an inflammatory marker.

### Reproduction

Lipocalin-type prostaglandin D_2_ synthase is involved in reproduction in both female and male genital organs. Serum L-PGDS levels are similar between pregnant and non-pregnant women (Shiki et al., 2004). L-PGDS levels are higher in the umbilical cord blood and amniotic fluid newborn urine than the maternal blood. L-PGDS levels in the cervicovaginal fluid are higher in patients with rupture of membrane than that without rupture of membrane (Shiki et al., 2004). In the normal pregnant women, urinary L-PGDS levels are higher in the third trimester than earlier pregnancy, while the plasma levels remain unchanged. Urinary L-PGDS levels are higher in early onset of preeclampsia as compared with late onset and in the severe compared to mild preeclampsia (Kinoshita et al., 2014). Urinary L-PGDS levels are a potential diagnostic marker for preeclampsia. L-PGDS levels in cervicovaginal secretion are two-fold higher in preterm births than full term births and inversely correlate against the days to expected delivery (Kumar et al., 2015). DP and CRTH2 antagonists may represent novel tocolytic agents for the treatment of preterm birth.

Patients with azoospermia show seminal plasma L-PGDS levels lower in oligozoospermic group than those in normozoospermic group (Tokugawa et al., 1998). L-PGDS levels in seminal plasma significantly reduce in severe oligozoospermic subfertile patients (Leone et al., 2001), and positively correlate with the alpha-glucosidase activity (Chen et al., 2005). Seminal plasma levels of L-PGDS are lower in patients with obstructive azoospermia than those with normal semen parameters, after vasectomy, or with nonobstructive azoospermia (Heshmat et al., 2008).

## Future Subjects

In the last two decades, more than 200 papers reported the multifunctional properties of L-PGDS. New reports are publishing almost every month in a variety of pathological and physiological function. To further accelerate understanding the function of the L-PGDS/PGD_2_ system, we still need several research probes as follows:

1) CRTH2-flox mice to make cell- or tissue-selective conditional KO mice.
2) L-PGDS-selective inhibitors with high affinities to study the L-PGDS-selective function *in vivo* pharmacologically.
  To understand the production of PGD_2_ within the cell, it remains unclear
3) How Cox and L-PGDS couple to each other topologically to achieve the efficient conversion from arachidonic acid, via unstable intermediate PGH_2_, to produce PGD_2_.
  To demonstrate the intercellular transport of L-PGDS and its ligands, we need the data of
4) LDL receptor-mediated internalization of L-PGDS in any types of cells.
5) L-PGDS-selective receptors to mediate the internalization of L-PGDS.
  Moreover, novel promising animal models are proposed as follows:
6) APP multiple mutant mice without L-PGDS may be a new animal model of Alzheimer’s disease with early accumulation of Aβ plaques.
7) Double KO mice of *L-Pgds* and *Hpgds* genes are new model animals of aging, because they show progressive age-related cartilage degradation (Ouhaddi et al., 2020) and adenomyosis (Philibert et al., 2021).

## Author Contributions

The author confirms being the sole contributor of this work and has approved it for publication.

## Conflict of Interest

The author declares that the research was conducted in the absence of any commercial or financial relationships that could be construed as a potential conflict of interest.

## Publisher’s Note

All claims expressed in this article are solely those of the authors and do not necessarily represent those of their affiliated organizations, or those of the publisher, the editors and the reviewers. Any product that may be evaluated in this article, or claim that may be made by its manufacturer, is not guaranteed or endorsed by the publisher.
