# Supplementary material for: Biochemical and Structural Characteristics, Gene Regulation, Physiological, Pathological and Clinical Features of Lipocalin-Type Prostaglandin D2 Synthase as a Multifunctional Lipocalin
Source: Front Physiol. 2021 Oct 22;12:718002. doi: 10.3389/fphys.2021.718002 (PMC8569824; doi:10.3389/fphys.2021.718002)
Supplement: Supplementary file 1 [file Table_1.pdf]

Table 1. History in the study of PGD_2 and L-PGDS

Year	Event	Ref
1961	Discovery of beta-trace as a major human CSF protein	Clausen 1961
1979	Purification of H-PGDS from rat spleen	Christ-Hazelhof and Nugteren. 1979
1982	Discovery of sleep induction by PGD_2 administration to rat brain	Ueno et al., 1982
1985	Purification of L-PGDS from rat brain	Urade et al., 1985
1987	Comparison between L-PGDS and H-PGDS as an example of functional convergence	Urade et al., 1987
1989	cDNA cloning of rat L-PGDS/Identification of N-terminal signal sequence & N-glycosylation site	Urade et al., 1989
1991	cDNA cloning of human L-PGDS/Characterization as a member of lipocalin	Nagata et al., 1991
1992	Chromosomal gene cloning of rat L-PGDS	Igarashi et al., 1992
1993	Dominant localization of L-PGDS in leptomeninges (arachnoid membrane) of the rat brain	Urade et al., 1993
	Identification of beta-trace as L-PGDS	Hoffmann et al., 1993
1994	cDNA cloning of DP receptor	Hirata et al., 1994
1995	Identification of Cys65 as an active site of L-PGDS	Urade et al., 1995
1997	Retinoid binding properties of L-PGDS	Tanaka et al., 1997
	cDNA cloning and crystallographic structure of rat H-PGDS	Kanaoka et al., 1997
	High expression of human L-PGDS in the heart and secetion of L-PGDS to coronary circulation	Eguchi et al;, 1997
	Generation of FP KO mice	Sugimoto et al., 1997
1998	Purification of L-PGDS from human seminal plasma/distribution of L-PGDS inhuman male organ	Tokugawa et al., 1998
1999	Generation of L-PGDS KO mice	Eguchi et al., 1999
	Identification of CRTH2 receptor as a PGD_2 receptor	Nagata et al., 1999
2000	Generation of human L-PGDS-overexpressing TG mice	Fujitani et al., 2000
	Generation of DP KO mice	Matsuoka et al., 2000
2001	cDNA cloning of CRTH2 receptor	Hirai et al., 2001
2006	Identification of HQL79 as an competitive inhibitor of H-PGDS	Aritake et al., 2006
	Generation of CRTH2 KO mice	Satoh et al., 2006
2007	Determination of NMR structure of mouse L-PGDS (Cys65Ala mutant)	Shimamoto et al., 2007
	Identification of L-PGDS as a major amyroid beta chaperon in CSF	Kanekiyo et al., 2007

	Generation of H-PGDS KO mice	Park et al., 2007
2009	Determination of X-ray crystallographic structure of mouse L-PGDS (Cys65Ala mutant)	Kumasaka et al., 2009
	Identification of AT56 as an competitive inhibitor of L-PGDS	Irikura et al., 2009
2010	Generation of human H-PGDS-overexpressing TG mice	Fujitani et al., 2010
	Determination of X-ray crystallographic structure of human L-PGDS	Zhou et al., 2010
2012	Generation of L-PGDS flox mice	Kaneko et al., 2012
2014	Determination of X-ray crystallographic structure of apo- and holo-forms of human L-PGDS	Perduca et al., 2014
2016	Generation of DP flox mice	Kong et al., 2016
2017	Generation of H-PGDS flox mice	Nakamura et al., 2017
2018	Generation of FP flox mice	Wang et al., 2018
2021	Substrate-induced product release mechanism of L-PGDS	Shimamoto et al., 2021
