# Supplementary material for: Biochemical and Structural Characteristics, Gene Regulation, Physiological, Pathological and Clinical Features of Lipocalin-Type Prostaglandin D2 Synthase as a Multifunctional Lipocalin
Source: Front Physiol. 2021 Oct 22;12:718002. doi: 10.3389/fphys.2021.718002 (PMC8569824; doi:10.3389/fphys.2021.718002)
Supplement: Supplementary file 2 [file Table_2.pdf]

Table 2. **Catalytic and molecular properties of human L-PGDS and H-PGDS**

	L-PGDS/β-trace	H-PGDS
Molecular weight of monomer	~26 000	~23 000
Tertiary structure	Monomer	Homodimer
Cofactor	Sulfhydryl compounds, such as dithiothreitol, β-mercaptoethanol, glutathione, cystein, cysteamine	Glutathione
Amino acid residues	190 (168 without signal sequence)	199
Activated thiol as an active site	Cys65	Glutahione bound to the enzyme
Inhibitor	SeCl4, AT56	HQL79, TFC007, TAS204
Activator		Mg^{2+}
Gene name	ptgds	hpgds
Chromosomal localization	9q34.3 (Chr19: 136.98 Mb)	4q22.3
Post-translational modification	N-glycosylation (Asn29 and Asn56), a core-1-HexHexNAc-O-glycan at Ser7, acetylation at Lys16 and Lys138, sulfonation at Ser41 and Thr142, and dioxidation at Cys43 and Cys145 witout signal sequence.	unknown
Subcellular localization	ER membrane, extracellular space (β-trace: CSF, inter-photoreceptor matrix, seminal plasma, plasma)	Cytosol (resting state), nuclear and ER membranes (active state after membrane trafficking)
