# Supplementary material for: Biochemical and Structural Characteristics, Gene Regulation, Physiological, Pathological and Clinical Features of Lipocalin-Type Prostaglandin D2 Synthase as a Multifunctional Lipocalin
Source: Front Physiol. 2021 Oct 22;12:718002. doi: 10.3389/fphys.2021.718002 (PMC8569824; doi:10.3389/fphys.2021.718002)
Supplement: Supplementary file 3 [file Table_3.pdf]

Table 3. Human genes, coupled G-proteins, ligands and physiological function of 3 distinct types of PGD_2 receptors.

PGD_2 receptors	DP	CRTH2	FP
**Other names**	DP1	DP2, GPR44, CD294	
**Gene**	ptgdr	ptgdr2	ptgfr
**Human chromosome**	Chr 14:52.27-52.28 Mb	Chr 11:60.85-60.86 Mb	Chr 1:78.3-78.54 Mb
**Coupled G protein**	Gs	Gi	Gq
**Agonists**	BW245C	13,14-dihydro-15-keto PGD_2	$PGF_{2\alpha}$, AL8810
**Antagonists**	BWA868C	Cay10471	Fluprostenol
	ONO4127Na	OC000459	
**Physiological function**	Regulation of sleep, pain, food intake, mast cell maturation Sertoli cell differentiation, and others.	Myelination of PNS, chronic stress-induced depression, adipocyte differentiation, inhibition of hair follicle neogenesis and others.	Dexamethasone-induced cardioprotection.
