# Supplementary material for: Biochemical and Structural Characteristics, Gene Regulation, Physiological, Pathological and Clinical Features of Lipocalin-Type Prostaglandin D2 Synthase as a Multifunctional Lipocalin
Source: Front Physiol. 2021 Oct 22;12:718002. doi: 10.3389/fphys.2021.718002 (PMC8569824; doi:10.3389/fphys.2021.718002)
Supplement: Supplementary file 4 [file Table_4.pdf]

Table 4. Non-substrate ligands of L-PGDS/β-trace

Ligand	Affinities Kd	Binding ratio	Methods*	Ref
**Retinoids**				
All-trans- or *9-cis*-retinoic acid	70-80 nM	1:1	TFQ, CD	Tanaka et al., 1997
All-trans- or *13-cis*-retinal				
All-trans-retinoic acid			NMR	Shimamoto et al., 2007
All-trans-retinoic acid,			SAXS	Inoue et al., 2009
**Thyroids**				
L-thyroxine	660 nM		TFQ, SPR	Beuckmann et al., 1999
3-3',5'-Triiodo-L-thyronine	820 nM			
3-3',5-Triiodo-L-thyronine	2.6 μM			
**Heme and its degradation products**				
Biliverdin	33 nM	1:1	TFQ, SPR	Beuckmann et al., 1999
Bilirubin	37 nM			
Bilirubin, biliverdin			SAXS	Inoue et al., 2009
Biliverdin			SAXS, NMR	Miyamoto et al., 2009
Biliverdin in human CSF	conjugates		NMR	Inui et al., 2014
Heme			NMR	Phillips et al., 2020
**Amyroid β (Aβ) peptides**				
Aβ(1-40), Aβ(!-42)	18-50 nM	1:1	SPR	Kanekiyo et al., 2007
Aβ(1-40), Aβ(25-35)		1:1	NMR, SAXS	Kannaian et al., 2019
**Lipids and fatty acids**				
GM1 and GM2 gangliosides	65-210 nM		SPR	Mohri et al., 2006a
Oleic acid & palmitoleic acid		1:(1+1)	X-ray crystallography	Zhou et al., 2010
Oleic acid	Ki=1.8 μM		Displacement of DAUDA binding, TFQ	Elmes et al., 2018
Palmitoleic acid	Ki=3.7 μM			
Synthetic FABP inhibitor SBFI-26	Ki=3.0 μM			
**Poorly water-soluble drugs**				
Diazepam, NBQX		1:3	ITC	Fukuhara et al., 2012
Telmisartan				Mizoguchi et al., 2015
Anti-cancer drug SN-38	60 μM	1:3	ITC	Nakatsuji et al., 2015
Telmisartan and imatinib	0.4-40 μM	1:2	ITC	Teraoka et al., 2017
Cannabinoid receptor 2 antagonists				
AM630	N.D.		Docking simulation	Yeh et al., 2019
WIN55212-2	N.D.			
**Cannabinoid metabolites, synthetic cannabinoids**				
Cannabidiol	Ki=78 μM		Displacement of DAUDA binding, TFQ	Elmes et al., 2018
Δ^9-tetrahydrocannabinol (THC)	Ki=175 μM			
11-hydroxy-Δ^9-tetrahydrocannabinol (11-OH-THC)	Ki=61 μM			
11-nor-9-carboxy-Δ^9-tetrahydro-cannabinol (THC-COOH)	Ki=7.8 μM			
Synthetic FABP inhibitor SBFI-26	3.0 μM			
**Anticholinergic drugs**				
Chlorpheniramine	4.3 - 5.7 μM	1:3	TFQ, ITC, NMR	Low et al., 2020
Trazodone	5.3 - 6.5 μM	1:3.4		

**Nicotinamide coenzymes**<br>NADPH, NADP+, and NADH	47-370 μM	1:1	ITC, NMR	Qin et al., 2015
**Substrate analog and product**<br>U-46619<br>PGD_2<br>PGE_2<br>PGF_{2a}	0.53 and 7.91 μM<br>0.31 and 44.3 μM<br>25.8 μM<br>2.52 and 137.4 μ M	1:2<br>1:2<br>1:1<br>1:2	ITC, NMR	Shimamoto et al., 2021

*TFQ: tryptophan fluorescence quenching, SAXS: Small angle X-ray scattering, SPR: Surface plasmon resonance,

ITC: Isothermal titration calorimetry, DAUDA: 11-(dansylamino)undecanoic acid.
