# Supplementary material for: Biochemical and Structural Characteristics, Gene Regulation, Physiological, Pathological and Clinical Features of Lipocalin-Type Prostaglandin D2 Synthase as a Multifunctional Lipocalin
Source: Front Physiol. 2021 Oct 22;12:718002. doi: 10.3389/fphys.2021.718002 (PMC8569824; doi:10.3389/fphys.2021.718002)
Supplement: Supplementary file 5 [file Table_5.pdf]

Table 5. Cell culture studies of L-PGDS

Cells and tissues	Species	Data	Ref
1) L-PGDS studies in cells of the central nervous system (CNS)			
Neuronal GT1-7 cells	Mouse	Dexamethasone induces L-PGDS gene transcription. TPA inhibits its induction.	García-Fernández et al., 2000
Primary cultured leptomeningeal cells	Rat	L-PGDS gene expression is down regulation by the Notch-Hes signal through the E-box and upregulated by IL-1β through the NF-κB pathway.	Fujimori et al., 2003
		Contact with astrocytes increases L-PGDS expression.	Fujimori et al., 2007b
Brain-derived TE671 cells	Human	L-PGDS expression is repressed by the Notch-Hes signal and maintained by AP-2β function. Protein kinase C activates L-PGDS expression through derepression of Notch-HES signal and enhancement of AP-2β function.	Fujimori et al., 2005
		L-PGDS gene expression is activated by USF-1 through the atypical E box within intron 4 and cooperatively by AP-2β in the promoter.	Fujimori and Urade. 2007
		L-PGDS gene expression is activated by USF-1 through the atypical E box within intron 4 through activation of p38MAPK by serum starvation	Fujimori et al., 2008
Neuroblastoma SH-SY5Y	Human	L-PGDS protects the neuronal cell death induced by oxidative stress.	Fukuhara et al., 2012
		L-PGDS prevents the paraquat-induced apoptosis via the NF-kB element in the proximal promotor region of the gene.	Fujimori et al., 2012
U251 glioma cell line	Human	Estradiol evokes an inverted U response of L-PGDS promoter activity in the cells expressing estrogen receptor (ER) α, but not β.	Devidze et al., 2010
Primary cultured astrocytes, microglia, cerebral endothelial cells, NIH3T3 fibroblast cells.	Mouse	L-PGDS activates glial cell migration and morphology as PGD_2-independent effects.	Lee et al., 2012
2) L-PGDS studies in vascular cells			
Vasular endothelial cells (ECs)	Human	Fluid shear stress upregulates L-PGDS expression.	Taba et al., 2000
		Fluid shear stress induces L-PGDS gene expression by transcriptional activation through the AP-1 site.	Miyagi et al., 2005
		Fluid shear stress increases L-PGDS expression in ECs of the CC genotype of the T786C single nucleotide polymorphism of the NO synthase 3.	Urban et al., 2019
Vascular smooth muscle cells from the aortic media	Rats	L-PGDS induces apoptosis of these cells. The apoptotic activity is reduced by site-directed mutagenesis in a glycosylation site Asn51, a protein kinase C phosphorylation site Ser106 and the enzyme active site Cys65.	Ragolia et al., 2007
3) L-PGDS studies in adipocytes			
Adipocytic 3T3-L1 cells	Mouse	L-PGDS is involved in adipocyte differentiation. L-PGDS gene expression is activated by LRH-1 in preadipocytes and by SREBP-1c in adipocytes.	Fujimori et al., 2007a
		Gene knockdown of L-PGDS by antisence L-PGDS in preadipocytes stimulates fat storage during the maturation stage.	Chowdhury et al., 2011
		Δ^{12}-PGJ_2, a dehydrated product of PGD_2, activates adipogenesis through PPARγ-dependent and independent pathways	Fujimori et al., 2012
		CRTH2 receptors are expressed in adipocytes and suppress the lipolysis by repression of the cAMP-protein kinase A-hormone sensitive lipase axis.	Wakai et al., 2017

		Glucocorticoid induces L-PGDS and leptin expression.	Yeh et al., 2019
Mesenchymal stroma cells	Human	siRNA-mediated downregulation of L-PGDS reduces adipocyte differentiation.	Lange et al., 2012
4) L-PGDS studies in skeltal muscle cells			
Skeltal muscle cell line L6	Rat	L-PGDS stimulates glucose transport.	Ragolia et al., 2008
5) L-PGDS studies in cells of the peripheral nervous system			
Primary culture of the dorsal root ganglion (DRG) neurons	Rat	L-PGDs is upregulated in DRG neurons by neuregulin 1(NRG) type III, a member of the NRG family of growth factors, and involved in myelination of Schwann cells by stimulation of CRTH2 receptors.	Trimarco et al., 2014
Primary culture of the enteric nervous system	Rat	L-PGDS is expressed in neurons and glia of the enteric cells and upregulated by LPS stimulation.	Le Loupp et al., 2015
6) L-PGDS studies in macrophages			
Macrophage cell line RAW 264.7 cells	Mouse	L-PGDS expression is upregulated by LPS or Pseudomonas, and regulated positively by AP-1 but negatively by p53.	Joo et al., 2007
		L-PGDS is induced by LPS through a transcription factor PU.1 activation by casein kinase II or NF-κB-inducing kinase, cooperated with MAPK-activated cJun.	Joo et al., 2009
7) L-PGDS studies in condrocytes			
Cultured osteoarthritic condrocytes	Human	L-PGDS is upregulated by IL-1β through the JNK and p38 MAPK and NF-κB signaling pathway	Zayed et al., 2008
8) L-PGDS studies in mast cell maturation			
Bone marrow-derived mast cells (BMMC)	Mouse	BMMC from KO mice of L-PGDS, DP, phospholipase A2 group 3 exhibit impaired maturation and anaphylaxis.	Taketomi et al., 2013
9) L-PGDS studies in cells of the skin			
Melanocytes, B16 melanoma cells	Mouse	L-PGDS is regulated by microphthalmia-associated transcription factor (MITF) responsible for malanocyte differentiation.	Takeda et al., 2006
Keratinocytes from neonatal foreskin	Human	L-PGDS inhibitor AT-56 counteracts the antimycotic-induced suppression of thymic stromal lymphopoietin production and the NF-κB activity.	Hau et al., 2013
10) L-PGDS studies in cancer cells			
Gastric cancer cell lines MKN45, HGC-27, MGC80-3 and 293 T cells	Human	L-PGDS and CRTH2 expression is suppressed by overexpression of Yesassociated protein 1 (YAP) in these cells. Overexpression of L-PGDS or CRTH2 decreases proliferation and self-renewal of these cells by YAP.	Bie et al., 2020
Cervical carcinoma cells	Human	L-PGDS knockdown by siRNA prevents chemotherapeutics-induced apoptosis.	Eichele et al., 2008
11) L-PGDS studies in cells of the prostate gland			
Prostate epithelial cells and fibroblasts	Human	L-PGDS and Cox-2 are induced in both cell types by low dose of bisphenolA. Inhibitors of L-PGDS (AT56) or Cox-2 (NS398) induce apoptosis of both cell types.	Wu et al., 2020
12) L-PGDS studies in sperm and oocytes			
Sperm and/or oocytes	Cow	Pretreatment of sperm and/or oocytes with antibody against L-PGDS inhibits in vitro fertilization and increases sperm-oocyte binding	Gonçalves et al., 2008a

Oocytes		Antibody against L-PGDS reacts with oocytes incubated with oviduct fluid and inhibits sperm binding, fertilization and embryonic development.	Gonçalves et al., 2008b
13) Amphibian ortholog of L-PGDS in Xenopus cells and embryo			
Xenopus A6 cells	Xenopus	Expression and characterization of amphibian L-PGDS	Irikura et al., 2007
Embryo		Xenopus L-PGDS participates in the establishment of the pre-placodal region of embryo as a retinoic acid transporter.	Jaurena et al., 2015
14) Intracellular interaction of L-PGDS and DP receptors in HEK and HeLa cells			
HEK cells, HeLa cells	Human	L-PGDS interacts intracellularly with DP in an agonist-independent manner and promotes cell surface expression of DP. DP export to the cell surface is dependent on DP–Hsp90 complex formation and the interaction between LPGDS and the C-terminal MEEVD residues of Hsp90. PGD2 synthesis by LPGDS is promoted by coexpression of DP.	Binda et al., 2014
HeLa cells		L-PGDS regulates the trafficking of DP by interacting with GTPase Rab4.	Binda et al., 2019
