# Supplementary material for: Biochemical and Structural Characteristics, Gene Regulation, Physiological, Pathological and Clinical Features of Lipocalin-Type Prostaglandin D2 Synthase as a Multifunctional Lipocalin
Source: Front Physiol. 2021 Oct 22;12:718002. doi: 10.3389/fphys.2021.718002 (PMC8569824; doi:10.3389/fphys.2021.718002)
Supplement: Supplementary file 6 [file Table_6.pdf]

Table 6. Animal experiments used for the study of L-PGDS

Animals	Phenotypes	Ref
**1) Central nervous system**		
**1-i) Pain response**		
L-PGDS-KO mice	Lack of PGE_2 or bicuculline-induced allodynia (touch-evoked pain)	Eguchi et al., 1999
Rats with lumbar disc herniation (LDH)	Overexpression and knockdown of L-PGDS, respectively, attenuates and worsens the herniation-triggered thermomechanical allodynia and degradation of DRG.	Xu et al., 2021
**1-ii) Sleep regulation**		
Human L-PGDS-overexpressing TG mice	Sleep attack after tail cutting.	Pinzar et al., 2000
L-PGDS or DP KO mice	Lack of non-rapid eye movement sleep rebound after sleep deprivation	Hayaishi et al., 2004
L-PGDS, H-PGDS, L/H-PGDS double, DP or CRTH2 KO mice	Lack of SeCl4-induced insomnia.	Qu et al., 2006
	L-PGDS, L/H-PGDS double or DP KO mice do not induce postictal sleep after seizure induced by pentylenetetrazole.	Kaushik et al., 2014
Leptomeninges-, oligo-dendrocytes-, or choroid plexus-specific L-PGDS KO mice	SeCl4-induced insomnia is lacked in leptomeninges-specific L-PGDS KO mice but found in oligodendrocytes- or choroid plexus-specific KO mice.	Cherasse et al., 2018
Estradiol-administered mice	L-PGDS expression is increased in the arcuate and ventromedial nucleus of the medial basal hypothalamus, a center of neuroendocrine secretions, and reduced in the ventrolateral preoptic area, a sleep center.	Mong et al., 2003
Ovarectomized female mice	Estrogen reduces L-PGDS expression in a region-dependent manner in the preoptic area, a sleep center. Estradiol benzoate induces high motor activity and reduces LPGDS mRNA in sleep-active areas.	Ribeiro et al., 2009
**1-iii) Neuronal and glial protection**		
L-PGDS and galactosylceramidase (GALC) double KO mice	L-PGDS is progressively upregulated in oligodendrocytes of GALC-deficient twitcher mice. In L-PGDS and GALC double KO mice, many neurons and oligodendrocytes show apoptosis.	Taniike et al., 2002
Lysosomal storage disorders mouse models	L-PGDS mRNA and immunoreactivity are upregulated in oligodendrocytes in these lysosomal storage disorders mouse models of Tay–Sachs disease, Sandhoff disease, GM1gangliosidosis and Niemann–Pick type C1 disease.	Mohri et al., 2006a
Neonatal mice	L-PGDS acts as an early stress protein to protect against neuronal death in the hypoxic ischemic encephalopathy.	Taniguchi et al., 2007
L-PGDS-KO mice	L-PGDS KO mice exhibit greater infarct volume and brain edema after cerebral ischemia than wild type mice.	Saleem et al., 2009
10-day -old rats	Intracerebroventricular (icv) administration of dexamethasone upregulates L-PGDS expression in the brain and protects neonatal hypoxic-ischemic brain injury.	Gonzalez-Rodriguez et al., 2014
Adult male Wistar rats	Chronic intermittent hypoxia (12h/day for 6 weeks) increases PGD_2 content and L-PGDS protein and mRNA from the 2nd week.	Shan et al., 2017
COX-2 and APP/PS1 crossed mice	L-PGDS expression is more enhanced in COX-2-overexpressing APP/PS1 mice than APP/PS-1 mice.	Guan et al., 2019
**1-iv) Amyloid β (Aβ) fibril prevention**		
L-PGDS-KO mice	L-PGDS KO mice increase deposition of Aβ(1-42) in the brain.	Kanekiyo et al., 2007
Human L-PGDS-overexpressing TG mice	L-PGDS TG mice decrease deposition of Aβ(1-42) in the brain.	
**1-v) Depression-related behavior**		

CRTH2-KO mice	CRTH2 KO or CRTH2 antagonist ramatoroban rescues the depression behavior induced by chronic treatment of corticosterone, lipopolysaccharide and tumor.	Onaka et al., 2015
**1-vi) Circadian rythm of behavior**		
L-PGDS-KO mice	L-PGDS KO mice and CRTH2 antagonst impair light-induced phase advance.	Kawaguchi et al., 2020
**1-vii) Food intake**		
Male ddY mice	L-PGDS is upregulated in hypothalamus after fasting. I.c.v. administration of PGD2 or DP agonist stimulates food intake. The orexigenic effect is blocked by DP and neuropeptide Y receptor antagonist.	Ohinata et al., 2008
L-PGDS-KO mice, DP KO mice, L-PGDS^{flox}/Nes-Cre mice	Orally administered rubiscolin-6, a δ opioid peptide, stimulates food intake in LPGDS^{flox}/Nes-Cre mice.	Kaneko et al., 2012
L-PGDS-KO mice	Activation of central δ-opioid receptor stimulates normal diet intake mediated by LPGDS but suppresses high-fat diet intake in an L-PGDS-independent manner.	Kaneko et al., 2014
**2) Myelination of peripheral nervous system**		
L-PGDS KO mice	L-PGDS KO mice have hypomyelinated peripheral nervous system.	Trimarco et al., 2014
L-PGDS KO mice	In injured peripheral nerves, L-PGDS controls macrophages accumulation in injured nerves, allowing myelin debris clearance and favoring axonal regeneration and remyelination.	Forese et al., 2020
**3) Lung inflammation**		
Human L-PGDS-overexpressing TG mice	L-PGDS TG mice show pronounced eosinophilic lung inflammation and Th2 cytokine release in ovalbumin-induced asthma model.	Fujitani et al., 2002
Human L-PGDS-overexpressing TG mice	L-PGDS TG mice show improved clearance of Pseudomonas from the lung.	Joo et al., 2007
L-PGDS-KO mice	L-PGDS KO mice show impaired clearance of Pseudomonas from the lung.	
H-PGDS KO mice	L-PGDS inhibitor AT56 suppresses accumulation of eosinophils and monocytes in the broncho-alveolar lavage fluid of antigen-induced inflammation.	Irikura et al., 2009
L-PGDS-KO mice	Lung edema is enhanced in L-PGDS KO mice in a HCl-induced acute lung injury model by a decrease in DP-mediated barrier function of endothelial cells.	Horikami et al., 2019
**4) Cardiovascular function**		
Estrogen-administered mice	L-PGDS is an estrogen receptor beta-regulated gene in the heart.	Otsuki et al., 2003
Heme oxygenase (HO) 2 KO mice	L-PGDS is induced in the heart by hypoxemia in wild-type mice and more significantly in HO-2 KO mice.	Han et al., 2009
L-PGDS-KO mice	L-PGDS KO mice exhibit reduction of dexamethasone-induced cardioprotection effects against ischemia/reperfusion injury.	Tokudome et al., 2009
FP KO mice, erythroid-derived 2–like 2 (Nrf2) KO mice	L-PGDS-mediated, dexamethasone-induced cardioprotection effects are not found in Nrf2-KO or FP KO mice.	Katsumata et al., 2014
Isolated perfused hypotoxic beeting rat atria	Hypoxia increases hypoxia-induced factor 1α (HIF-1α), stimulates ANP secretion and upregulates L-PGDS. HIF-1α antagonist attenuates hypoxia-induced ANP secretion and down regulates L-PGDS level. L-PGDS inhibitor AT-56 attenuated hypoxia-induced ANP secretion.	Li et al., 2018
	Acute hypoxia increases expression of L-PGDS and Cox-2 through endothelin (ET)-1 secretion and activation of ET_A and ET_B receptors.	Li et al., 2019
**5) Obesity and adipocyte differentiation**		

L-PGDS-KO mice	L-PGDS KO mice exhibit glucose-in-tolerant and insulin-resistant at an accelerated rate, and larger adipocytes than wild-type mice. L-PGDS KO mice develop nephropathy and an aortic thickening reminiscent to the early stage of atherosclerosis when fed a diabetogenic high fat diet.	Ragolia et al., 2005
	L-PGDS KO mice exhibit reduced sensitivity to insulin-stimulated glucose transport.	Ragolia et al., 2008
	L-PGDS KO mice exhibit more severe atherosclerotic plaque in the aorta, macrophage cellularity and expression of pro-inflammatory responses than wild-type mice.	Tanaka et al., 2009
	L-PGDS KO mice show early elevations in hypothalamic-pituitary-adrenal activity, plasma corticosterone and ACTH concentrations.	Evans et al., 2013
L-PGDS KO mice, L-PGDS knock-in (KI) mice	L-PGDS KO mice decrease beneficial metabolic effects after vertical sleeve gastrectomy.	Kumar et al., 2016
L-PGDS-KO mice, H-PGDS KO mice	L-PGDS KO mice show hypertension and acceleration of thrombogenesis.	Song et al., 2018
L-PGDS^{flox}-fatty acid bind-ing protein 4 (aP2)-Cre mice; /L-PGD^{flox} adipo-nectin (AdipoQ)-Cre mice	High fat diet-induced body weight gain is reduced only in aP2-Cre/L-PGDS^{flox} mice with the premature adipocyte-selective deletion but unchanged in AdioQ-Cre/L-PGDS^{flox} mice with the mature adipocyte-selective deletion.	Fujimori et al., 2019
PPARγ-KO mice, PPARγ-LPGDS double KO mice	LPGDS is upregulated in brown and white adipose tissues of PPARγ-KO mice. PPARγ–LPGDS double KO mice reduce the expression of thermogenic genes. LPGDS and PPARγ coordinate to regulate carbohydrate and lipid metabolism.	Virtue et al., 2012b
LPGDS KO mice under cold acclimated conditions	LPGDS expression in brown adipose tissue (BAT) is correlated with the BAT activity of thermogenesis. Under cold-acclimated conditions, LPGDS KO mice elevate reliance on carbohydrates.	Virtue et al., 2012a
Wild-type C57BL/6 and ob/ob mice	LPGDS is down regulated in white adipose tissue of ob/ob mice, whereas HPGDS is upregulated in WAT of ob/pb mice.	Virtue et al., 2015
**6) Bone and cartilage metabolism**		
DBA/1Jmice	L-PGDS H-PGDS, DP, CRTH2 are upregulated in articular tissues during the arthritic process. The injection of PGD_2 or DP agonist into paw attenuates the incidence of collagen-induced arthritis, the inflammatory response and join damage.	Maicas et al., 2012
Hartley guinea pig, dog	L-PGDS increases over the course of osteoarthritis. L-PGDS levels correlated positively with the histological score of osteoarthritis.	Nebbaki et al., 2013
L-PGDS KO mice	L-PGDS KO mice exacerbate experimental osteoarthrisis.	Najar et al., 2020
	L-PGDS KO mice exhibit promoted cartilage degradation, enhanced subchondral bone changes without effect on its angiogenesis, increased mechanical sensitivity and reduced spontaneous locomotor activity during aging.	Ouhaddi et al., 2020
**7) Keratinocytes and hair follicle neogenesis**		
C57Bl/6J, FVB/N and Mixed (C57Bl/6J × FVB/N × SJL) mice, CRTH2-KO mice	L-PGDS and PGD_2 levels of the skin among these strains of mice are negatively correlated with wound-induced hair follicle neogenesis. CRTH2-KO mice, but not DP KO mice, increase the hair follicle regeneration. L-PGDS is expressed in keratinocytes in the skin.	Nelson et al., 2013
**8) Fibloblastic L-PGDS for mast cell maturation and anaphylaxis**		
L-PGDS KO mice, DP KO mice, phospholipase A2 group 3 (PLA2G3) KO mice, mast cell-deficient mice	PLA2G3 secreted from mast cells is coupled with fibroblastic L-PGDS to provide PGD2, which facilitates mast cell maturation via DP.	Taketomi et al., 2013
**9) Colon**		
L-PGDS-KO mice	L-PGDS KO mice improve dextran sodium sulfate-induced colitis.	Hokari et al., 2011

**10) Adenoma growth in intestine and colon**		
Apc$^{Min/+}$ mice mated with human H- or L-PGDS-TG and H-PGDS, L-PGDS or DP KO-mice	Overexpression of H-PGDS or L-PGDS in Apc$^{(Min/+)}$ mice have fewer adenoma and HPGDS or DP KO in APC$^{(Min/+)}$ mice, more adenoma.	Tippin et al., 2014
**11) Melanoma growth**		
L-PGDS-KO mice, endothelial cells (EC)-specific L-PGDS KO mice	Melanoma growth is accelerated in L-PGDS KO mice and EC-selective L-PGDS KO mice.	Omori et al., 2018
**12) Renal function**		
Adriamycin-induced nephropathy of mice	Urinary L-PGDS excretion increases before albuminuria.	Tsuchida et al., 2004
Otsuka Long-Evans Tokushima Fatty (OLETF) rats	Urinary secretion of L-PGDS is higher in OLETF rats than non-diabetic Long-Evans Tokushima Otsuka (LETO) rats, in an age-dependent manner.	Ogawa et al., 2006
Monkey	L-PGDS is produced de novo in the cells of Henle's loop and the glomeruli of the kidney and N-terminal truncated in the lysosomes of tubular cells.	Nagata et al., 2009
L-PGDS-KO mice, CRTH2-KO mice, IL4 KO mice, IL13 KO mice	Unilateral ureteral obstruction induces renal fibrosis, which is exacerbated by the LPGDS/CRTH2/Th 2 cytokines pathway.	Ito et al., 2012
**13) Preterm birth**		
L-PGDS-KO mice, Human L-PGDS overexpressing TG mice	Incidence of preterm birth by LPS administration is decreased in KO mice and increased in TG mice. DP and CRTH2 antagonists increase the number of viable pups.	Kumar et al., 2015
**14) Adenomyosis**		
L/H-PGDS double KO mice	L/H-PGDS double KO mice develop adenomyotic lesions at 6-month-old and the disease severity increases with age.	Philibert et al., 2021
**15) Testis and epididymis**		
Bull	L-PGDS is expressed in testis, epididymis and ejaculated sperm.	Gerena et al., 2000
Rats	L-PGDS expression is decreased after castration or ethylene dimethane sulfonate treatment and recovered by testosterone.	Zhu et al., 2004
L-PGDS-KO mice	Heterozygous and homozygous L-PGDS KO mice present unilateral cryptorchidism affecting the second phase of testicular descent in 16% and 24% of case, respectively.	Philibert et al., 2013
**16) Prostate gland**		
Male SD rats, bisphenol A-induced prostatic hyperplasia	Low dose bisphenol A upregulates L-PGDS expression in ventral prostate.	Wu et al., 2020
**17) Male germ cell differentiation in the foetal testis**		
MF1 mice	L-PGDS is identified as the male-enriched transcript in mouse embryonic gonads.	Adams and McLaren, 2002
Swiss mice	PGD_2 induces nuclear import of the sex-determining factor Sox9 via its cAMP-PKA phosphorylation mediated by DP receptors.	Malki et al., 2005
Swiss Quackenbush outbred mice	SOX9 binds to and activates the L-PGDS promoter during testis development.	Wilhelm et al., 2007
L-PGDS KO mice, Sox9 KO mice, Fgf KO mice	L-PGDS expression is down-regulated in Sox9 KO gonads, but unchanged by Fgf KO gonads. Both Fgf9 and PGD_2 signaling cooperate to upregulate Sox9 in Sertoli cells.	Moniot et al., 2009
L-PGDS KO mice, H-PGDS KO mice	An initial H-PGDS-mediated PGD_2 signal participates in the Sox9 nuclear translocation necessary for the process of Sertoli cell differentiation.	Moniot et al., 2011

L-PGDS, H-PGDS, L/H-PGDS double KO mice, DP, CRTH2 KO mice	The double KO mice and CRTH2 KO mice exhibit increased proliferation rates of germ cells without being mitotically arrested at the late phase of embryonic stages. Both somatic- and germ cell-produced PGD_2, acting in both a paracrine and an autocrine manner, plays a role in the regulation of male foetal germ cell differentiation through CRTH2 receptors.	Moniot et al., 2014
**18) Pharmacokinetics of L-PGDS**		
Dogs	After an i.v. bolus injection, the serum concentration L-PGDS decreases with t1/2 of 0.77 h, which is shorter than that of other protein with the same Mr. About 10% of LPGDS is secreted to the urine. After the intrathecal injection, about one third of the injected L-PGDS is excreted to the blood.	Li et al., 2008
**19) Functionalization of nanoparticles by L-PGDS**		
C57/BL mice	L-PGDS-conjugated nanoparticles are taken up by neurons and glial cells mediated by an LDLR-mediated mechanism after the administration from tail vein.	Portioli et al., 2017
**20) Nonmammalian ortholog of L-PGDS in birds**		
Chiken, Dorsal root ganglion	L-PGDS-immunoreactivity is localized in DRG neurons and H-PGDS, in DRG glia.	Vesin et al., 1995
Chiken, Brain, heart, gene	Chicken L-PGDS is associated with the enzyme activity and the binding activity of thyroxine and all-trans retinoic acid.	Fujimori et al., 2006
Chiken, Testis	L-PGDS is expressed in a male specific expression mechanism related with SOX9	Moniot et al., 2008
**21) Nonmammalslian ortholog of L-PGDS in amphibian**		
Xenopus, Cane toad, Japanese tree frog, genes	Identification of toad L-PGDS with the enzyme activity and the binding activity of thyroid hormones and all-trans retinoic acid.	Irikura et al., 2007
Xenopus, Placode progenitor formation	Xenopus L-PGDS participate in the establishment of the pre-placodal region.	Jaurena et al., 2015
**22) Nonmammallian ortholog of L-PGDS in fish**		
Zebrafish, Gene	Identification of Zebrafish L-PGDS without the enzyme activity yet with the binding activity of thyroxine and all-trans retinoic avid	Fujimori et al., 2006
Catfish, Seminal vesicle, testis	L-PGDS expression in seminal vesicle is decreased by L-thyroxin overdose and increased by depletion of thyroid hormone. Treatment of catfish with human chorionic gonadotropin and estradiol reduces the L-PGDS expression	Sreenivasulu et al., 2013
