# Supplementary material for: Biochemical and Structural Characteristics, Gene Regulation, Physiological, Pathological and Clinical Features of Lipocalin-Type Prostaglandin D2 Synthase as a Multifunctional Lipocalin
Source: Front Physiol. 2021 Oct 22;12:718002. doi: 10.3389/fphys.2021.718002 (PMC8569824; doi:10.3389/fphys.2021.718002)
Supplement: Supplementary file 7 [file Table_7.pdf]

Table 7. Human pathology of L-PGDS/beta-trace

Tissues and diseases	Phenotypes	Sampling numbers	Ref
**1) CNS**			
**1-1) Sleep regulation**			
Mastocytosis	Serum level of PGD_2 metabolite increases during sleep attack of patients with mastocytosis. The sleep attack is suppressed by aspirin but not prevented by antihistaminergic drugs.		Roberts et al., 1980
African sleeping sickness	CSF PGD_2 concentrations selectively and markedly elevate in the late stage patients.	24 patients, 12 patients without CNS involvement	Pentreath et al., 1990
Physiological sleep	Serum L-PGDS levels increase in evening and are suppressed by total sleep deprivation but unchanged by REM sleep deprivation.	20 healthy humans	Jordan et al., 2004
Excessive daytime sleepiness (EDS)	CSF L-PGDS levels are lower in EDS patients than control.	34 patients, 22 healthy controls	Bassetti et al., 2006
Narcolepsy	Serum levels of L-PGDS are higher in narcoleptic patients than healthy control and are correlated with the sleepiness scale.	14 narcolepsy, 14 healthy control	Jordan et al., 2005
Narcolepsy, ideopathic hypersomnia (IH)	CSF and serum levels of L-PGDS are higher in patients with narcolepsy and IH than control.	122 narcolepsy, 27 IH, 51 control	Wang et al., 2020
Obstructive sleep apnea (OSA)	Serum L-PGDS levels are higher in OSA patients with excessive daytime sleepiness (EDS) than those without EDS or controls.	47 patients with OSAS (26 with and 21 without EDS), 18 healthy controls	Barceló et al., 2007
	Urinary L-PGDS levels are higher in patients with severe OSA than those in control or moderate OSA. The urinary excretion of severe OSA patients is reduced to the control level after continuous positive airway pressure treatment.	23 severe OSA, 25 moderate OSA, 16 control,	Chihara et al., 2013
**1-2) Food intake**			

Food intake	CSF L-PGDS levels at the base line are correlated positively with neuropeptide Y (NPY), galanin and visceral adipose tissue, corticotropin-releasing hormone and beta-endorphin and inversely with CSF leptin. Leptin treatment does not affect CSF L-PGDS and NPY levels.	26 subjects in a weight loss study, comprising a 3-week dietary lead-in followed by 12-weeks of leptin or placebo treatment	Elias et al., 2011
**1-3) CSF circulation**			
Normal pressure hydrocephalus (NPH)	CSF L-PGDS levels are lower in NPH patients than control.	14 NPH patients, 14 control	Mase et al., 2003
	CSF L-PGDS levels decrease in disproportionately enlarged subarachnoid-space hydrocephalus.	22 NPH patients	Nishida et al., 2014
	CSF L-PGDS levels show a trend of increase in the cognitive-improved patients after lumbo-peritoneal shunting but not in the poor cognitive-improved patients.	60 NPH patients	Nakajima et al., 2015
Spontaneous intracranial hypotension (SIH)	CSF L-PGDS levels are higher in SIH patients than control.	62 patients (38 SIH, 24 non-SIH), 10 control	Murakami et al., 2018
CSF fluid drainage	The diploic vein/peripheral vein ratio of serum L-PGDS concentrations is high in the frontal, temporal, parietal and skull base. For patients older than 45 years, the diploic vein/peripheral vein ratio is reduced in the frontal region.	51 patients underwent 41 cranial and 10 spinal surgeries	Tsutsumi et al., 2015
**1-4) Neurodegenerative diseases**			
Multiple sclerosis (MS)	CSF L-PGDS levels are unchanged in patients with MS.	CSF collected at post mortem from 6 of 8 MS patients	Kagitani-Shimono et al., 2006
	L-PGDS immunoreactivity is increased in oligodendrocytes within the shadow plaques and in hypertrophied astrocytes within the chronic plaques of autopsy samples from MS patients. (The Human Brain and Spinal Fluid Resource Center (HBSFRC; Los Angeles, CA, USA))	Paraffin sections of brain tissues (5 MS and 3 non-MS controls), fresh frozen blocks (5 MS patients and 3 non-MS controls)	
Neonatal hypoxic ischemic encephalopathy (HIE)	L-PGDS immunoreactivity is detected in the surviving neurons in the infarcted lesions in autopsy samples from patients with HIE.	8 HIE patients, 8 age-matched control patients who had died from non-neurological diseases.	Taniguchi et al., 2007

Subarachnoid hemorrhage (SAH)	CSF L-PGDS levels increase after SAH.	6 patients with aneurysmal SAH	Mase et al., 1999
	CSF L-PGDS in patients after SAH binds billiverdin covalently and scavenges harmful heme-degradation products.		Inui et al., 2014
**2) Cardiovascular diseases**			
Angina	L-PGDS-immunoreactivity is expressed in myocardial cells, atrial endocardial cells and synthetic phenotype of smooth muscle cells of the heart of patients with angina.<br>Plasma L-PGDS level is higher in the great cardiac vein than the coronary artery.	7 patients with angina, 7 patients without angina	Eguchi et al., 1997
Restenosis after coronary angioplasty	Serum L-PGDS levels in coronary sinus blood remain to the baseline level in patients with restenosis but increase in those without restenosis at 48 hr after percutaneous transluminal coronary angioplasty (PTCA) .	24 patients undergoing PTCA	Inoue et al., 2001
Hypertension	Serum L-PGDS levels increase in hypertensive patients with the renal function worsened. The urinary excretion is higher in hypertensive patients than normotensive patients.	111 patients with hypertension, 102 normotensive subjects	Hirawa et al., 2002
Carotid atherosclerosis	In terms of a common SNP (4111 A>C in 3'-untranslated region) of the L-PGDS gene in Japanese, serum levels of HDL cholesterol are higher in the subjects with A/A genotype than those with A/C and C/C genotypes. The maximum intima-media thickness in the common carotid artery is smaller in subjects with A/A genotype than those with A/C and C/C.	782 Japanese hypertensive subjects	Inoue et al., 2004
Subclinical atherosclerosis	Serum L-PGDS levels increase in associated with the progression of atherosclerosis.	500 non-treated asymptomatic subjects	Miwa et al., 2008
Stable coronary artery disease	Serum L-PGDS levels are powerful biomarker of severity of stable coronary artery disease.	1013 patients with coronary angiography	Inoue et al., 2008
Coronary vasospasm	Serum L-PGDS levels are higher in patients with vasospastic angina, and negatively correlated with the degree of the left anterior descending coronary artery vasomotion in response to Ach.	96 patients with diagnostic coronary angiography	Matsumoto et al., 2011

Aterosclerosis	Serum L-PGDS levels are not different between 4 groups of (1) no previous cardiovascular disease (CVD) and no coronary calcification, (2) no previous CVD but coronary calcifications, (3) acute coronary syndrome, and (4) clinical stable patients with CVD.	120 gender- and age-matched individuals with or without cardiovascular disease	Hosbond et al., 2014
Pulmonary embolism	Venous blood L-PGDS levels are higher in patients with pulmonary embolism than control.	90 patients, 40 healthy volunteers	Mutlu et al., 2020
**3) Metabolic syndrome**			
Metabolic syndrome	Serum L-PGDS levels are associated with hypertriglyceridemia but not diabetes.	3136 patients	Cheung et al., 2013
**4) Renal function**			
Diabetes mellitus	Urinary L-PGDS excretion increases in the early stage of kidney injury in patients with type-2 diabetes mellitus.	36 type-2 diabetic patients, 29 normal subjects	Hirawa et al., 2001
	Blood sugar control reverses the increase in urinary excretion of L-PGDS in diabetic patients. L-PGDS is present in the renal tubules in diabetes patients but not in nondiabetic patients.	55 type-2 diabetic outpatients, 55 age-matched healthy control subjects	Hamano et al., 2002
	Urinary L-PGDS levels are significantly associated with cardiovascular diseases and may be a supplemental or additional marker to the criteria of metabolic syndrome.	233 Japanese type 2 diabetes patients	Yoshikawa et al., 2007
	Urinary L-PGDS levels reflect the current increased permeability of injured glomerular capillary wall and are better predictor of the future status of renal injury in type-2 diabetes with <30 mg/Cr albuminurea	793 healthy subjects, 200 patients with various forms of renal diseases, 666 patients with type-2 diabetes; In the prospective study, 121 type-2 diabetic patients with <30 mg/g Cr albumiurea for almost 2 years.	Uehara et al., 2009
Gentamycin-induced renal damage	Urinary L-PGDS excretion increases in patients with long-term administration of gentamycin	6 patients with long-term administration of gentamycin	Nakayama et al., 2009
Systemic lupus erythematosus (SLE), lupus nephritis (LN)	Urinary L-PGDS is significantly higher with active vs. inactive LN or in patients without LN. Urinary L-PGDS excretion increases as 3 months before a clinical diagnosis of worsening LN.	98 children with SLE, 30 control	Suzuki et al., 2009

Mucopolysaccharidosis type II (MPSII, Hunter disease)	Urinary L-PGDS is lower in MPSII patients.	12 MPS II patients, 12 (171)	Yuan et al., 2019
**5) Cancers**			
Ovarian cancer	L-PGDS is expressed in tumor cells of all various types of ovarian cancers.	54 ovarian cancer	Su et al., 2001
Lung tumors	L-PGDS expression is diminished in lung tumors		Ragolia et al., 2010
Melanoma	L-PGDS is overexpressed in malignant melanomas		Shimanuki et al., 2012
Gastric cancer (GC)	L-PGDS expression is negatively correlated with Yes-associated protein 1 (YAP) in GC.	60 paired GC tissues and adjacent tissues	Bie et al., 2020
**6) Bone and cartilage**			
Osteoarthritis (OA)	L-PGDS increases in cartilage of patients with OA.	13 autopsy from no history of OA, 32 samples OA patients under-going total knee replacement	Zayed et al., 2008
**7) Digestive tract**			
Helicobacter pyloriinduced gastritis	L-PGDS is induced on fibroblasts close to infiltrating cells in the H. pylori-infected gastric mucosa of biopsy samples.	60 patients	Hokari et al., 2009
Ulcerative colitis (UC)	L-PGDS is increased in lamina proprial infiltrating cells and muscularis mucosa in colonic biopsy from UC patients in parallel with the disease activity	24 patients with UC, the non-inflamed mucosa from 9 patients with UC, 16 patients with colonic polyps as controls	Hokari et al., 2011
Crohn's disease (CD)	L-PGDS and Cox-2 mRNA expressions and PGD_2 levels increase in inflamed colonic mucosa of colonic biopsies from patients with active CD. L-PGDS is expressed in neurons of both human myenteric and submucosal plexi.	30 patients with CD (15 quiescent CD and 15 active CD),<br>15 controls	Le Loupp et al., 2015
**8) Inflammation**			

Clinically healthy 58 years old Swedish men	Serum L-PGDS is not correlated with insulin sensitivity but positively with soluble TNF receptors 1 and 2 and negatively with alcohol consumption and serum HDL.	100 men were selected among 818 screened subjects	Wallenius et al., 2011
**9) Reproduction**			
Pregnancy, rupture of membrane (ROM)	Serum L-PGDS levels are similar between pregnant and non-pregnant women. LPGDS levels are higher in umbilical cord blood and amniotic fluid newborn urine than maternal blood. L-PGDS levels in cervicovaginal fluid are higher in ROM than that without ROM.		Shiki et al., 2004
Preeclampsia (PE)	Plasma and urinary L-PGDS levels are higher in PE patients than normal pregnant women.	36 PE patients, 94 normal pregnant women	Kinoshita et al., 2014
Preterm birth (PTB)	L-PGDS levels in cervicovaginal secretion are 2-fold higher in PTB than full term births and inversely correlated against the days to expected delivery.	370 pregnant women (296 PTB, 74 full term birth)	Kumar et al., 2015
Oligozoospamia	Seminal plasma L-PGDS levels are lower in oligozoospermic group than normozoospermic group.	10 oligozoospermic men, 41 normozoospermic men	Tokugawa et al., 1998
	Seminal plasma L-PGDS levels are significantly reduced in severe oligozoospermic subfertile patients.	59 adult males	Leone et al., 2001
	Seminal plasma L-PGDS levels are reduced in patients with azoospermia.	68 samples	Chen et al., 2005
	Seminal plasma L-PGDS levels are decreased in patients with obstructive azoospermia.	10 patients with normal semen parameters, 9 with obstructive azoospermia, 20 after vasectomy, 14 with non-obstructive azoospermia	Heshmat et al., 2008
